# N-of-1 Clinical Trials in Nutritional Interventions Directed at Improving Cognitive Function

**DOI:** 10.3389/fnut.2019.00110

**Published:** 2019-07-23

**Authors:** Natalia Soldevila-Domenech, Anna Boronat, Klaus Langohr, Rafael de la Torre

**Affiliations:** ^1^Integrative Pharmacology and Systems Neurosciences Research Group, Neurosciences Research Program, Hospital del Mar Medical Research Institute (IMIM), Barcelona, Spain; ^2^Department of Experimental and Health Sciences, University Pompeu Fabra, Barcelona, Spain; ^3^Department of Statistics and Operations Research, Universitat Politècnica de Barcelona/Barcelonatech, Barcelona, Spain; ^4^CIBER de Fisiopatología de la Obesidad y la Nutrición (CIBEROBN), Instituto de Salud Carlos III, Madrid, Spain

**Keywords:** N-of-1, personalized nutrition, prevention, Alzheimer's disease, multimodal interventions, cognition, cognitive decline

## Abstract

Longer life expectancy has led to an increase in the prevalence of age-related cognitive decline and dementia worldwide. Due to the current lack of effective treatment for these conditions, preventive strategies represent a research priority. A large body of evidence suggests that nutrition is involved in the pathogenesis of age-related cognitive decline, but also that it may play a critical role in slowing down its progression. At a population level, healthy dietary patterns interventions, such as the Mediterranean and the MIND diets, have been associated with improved cognitive performance and a decreased risk of neurodegenerative disease development. In the era of evidence-based medicine and patient-centered healthcare, personalized nutritional recommendations would offer a considerable opportunity in preventing cognitive decline progression. N-of-1 clinical trials have emerged as a fundamental design in evidence-based medicine. They consider each individual as the only unit of observation and intervention. The aggregation of series of N-of-1 clinical trials also enables population-level conclusions. This review provides a general view of the current scientific evidence regarding nutrition and cognitive decline, and critically states its limitations when translating results into the clinical practice. Furthermore, we suggest methodological strategies to develop N-of-1 clinical trials focused on nutrition and cognition in an older population. Finally, we evaluate the potential challenges that researchers may face when performing studies in precision nutrition and cognition.

## Introduction

Population aging has led to a substantial increase in the worldwide prevalence of dementia (1). Currently, around 50 million people live with this condition, a figure that is expected to triple by 2050 (2). Dementia comprises a wide range of medical and neuropsychiatric disorders. It is characterized by a progressive cognitive decline greater than expected in normal aging, strongly affecting the individual's daily living activities and quality of life (3). Alzheimer's disease (AD) is the most common cause of dementia, accounting for 50–70% of cases (2).

To date, there is a lack of effective disease-modifying therapies for dementia. Since prevention is always better than cure, interventions designed to prevent or delay the onset of dementia represent nowadays an immediate research priority (2). Attention has focused on modifiable risk factors, including obesity, hypertension, diabetes, and physical inactivity (2, 4). Evidence supports the fact that management of these conditions through lifestyle interventions, such as healthy diets and physical activity, may benefit cognitive function and reduce dementia risk and severity (5–8). Indeed, optimal nutrition is a key component for healthy aging. Nutritional preventive interventions present unique advantages in terms of costs, safety, and sustainability for long-term use (9, 10).

Although there is extensive literature discussing nutritional strategies for the prevention of dementia, current evidence presents inconsistent results. The heterogeneity of dementia from a clinical, pathophysiological, and genetic viewpoint suggests that dietary interventions are not universally applicable, but when tailored to individual circumstances and risk profiles might be more effective (8, 11, 12). Recently, the concept of precision medicine for the prevention and management of AD has emerged in the scientific community as a new model for obtaining solid evidence-based medicine (13). An approach that requires accounting for the inter-individual variability in treatment response.

The gold-standard study design for assessing the effectiveness of an intervention are the population-based, parallel-group, randomized-controlled trials (RCTs) (14). However, such effectiveness is commonly assessed using the average treatment effect or type of subgroup analysis, without effectively tackling the individual particular characteristics that may modify treatment response (15). In addition, conventional RCTs seek a high homogeneity within the study population in order to increase the likelihood of demonstrating a true association between intervention exposure and outcomes. As a result of the strict inclusion and exclusion criteria, there is a considerable lack of representativeness of some groups of patients, including those with comorbid conditions or receiving concurrent therapies (16, 17). As a result, evidence from RCTs cannot always be extrapolated in the case of individual treatment decisions (17). In the age of patient-centered care, a patient-oriented research is called for (18, 19).

N-of-1 trials are coming to light in the medical field to address the question of inter-individual variability in treatment response, and the lack of knowledge about treatment effects in patients who are typically excluded in RCTs. An N-of-1 trial refers to a randomized, multiple, crossover trial conducted in a single patient, typically where two or more treatment alternatives are compared to each other or to a control intervention (17, 20). It is noteworthy to mention that the Oxford Center for Evidence-Based Medicine 2011 Levels of Evidence has categorized N-of-1 trials as “level 1” of evidence for assessing treatment efficacy in individual patients (21). For the purpose of valid inference in individual patients, however, in N-of-1 trials, more data needs to be collected from the patient than in conventional RCTs, which focus on between patients rather than within patient variation.

N-of-1 trials are part of the single-case design family (22), which were first carried out in 1945 (23). Since then, they have been widely used in the study of rare diseases (24), in some medical areas such as pain, rheumatism and pediatric oncology (25–30), and in the psychology, social, and educational sciences. A renewed interest in this design has arisen combined with the emergence of precision medicine to tackle inter-individual differences within treatment responses. In parallel, the development of electronic health information technology (e.g., mobile apps and fitness trackers) has facilitated the implementation of tools to intensively follow-up study participants collecting data in a systematic and detailed way (31). Such information enables researchers to precisely determine the effect of an intervention at an individual level (32). Moreover, the development of analytical tools capable of processing all the generated data makes N-of-1 studies a feasible and realistic approach for future precision medicine. In addition, data aggregation from series of N-of-1 trials also makes it possible to estimate effects on a subpopulation that shares certain factors, or even in the population at large (12, 33).

**Figure 1:**
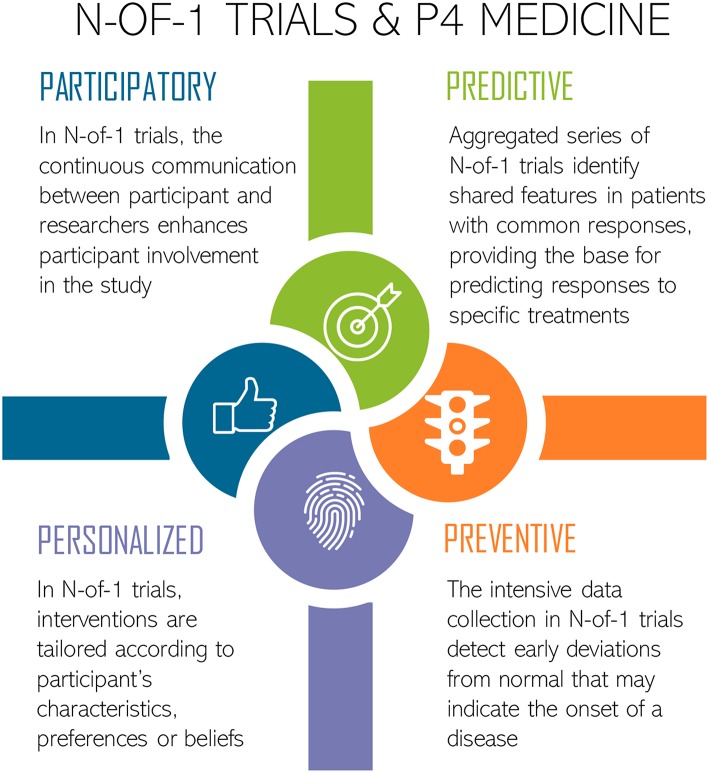
N-of-1 trials approach for P4 Medicine: Predictive, Preventive, Personalized, and Participatory. Icons made by Freepik from www.flaticon.com.

The holistic approach of N-of-1 studies would, therefore, be useful to manage P4 medicine—predictive, preventive, personalized, and participatory (Figure 1) (34–36). Firstly, however, through N-of-1 trials it is possible to obtain information of both between- and within-subject variations on biomarkers, which could play a key role in identifying those that are truly predictive (35). Secondly, intensive data collection over time enables the detection of deviations from the norm which may signal disease onset (35). Thirdly, it is possible to stratify treatments according to the combination of some patient features (e.g., gender, age, culture, socioeconomic status, history of diseases) and the molecular and general-*omics* profile, which is the basis of personalized or precision medicine (37, 38). Finally, in N-of-1 trials, patients are more likely to participate, as they may directly benefit from the tailored treatment (35).

## Dementia Prevention Through Nutritional Interventions

### Alzheimer's Dementia Continuum

Dementia is the clinically observable result of the accumulation of structural and functional cerebral damage that has commenced 10–20 years prior to the appearance of the first symptoms (39, 40). Whilst aging is the strongest risk factor, dementia is not an inevitable consequence. Environmental factors (e.g., biological, psychological, and lifestyle factors), genetic susceptibility, and their interaction over the life span contribute to the physiopathological processes and clinical manifestation of dementia (19).

**Figure 2:**
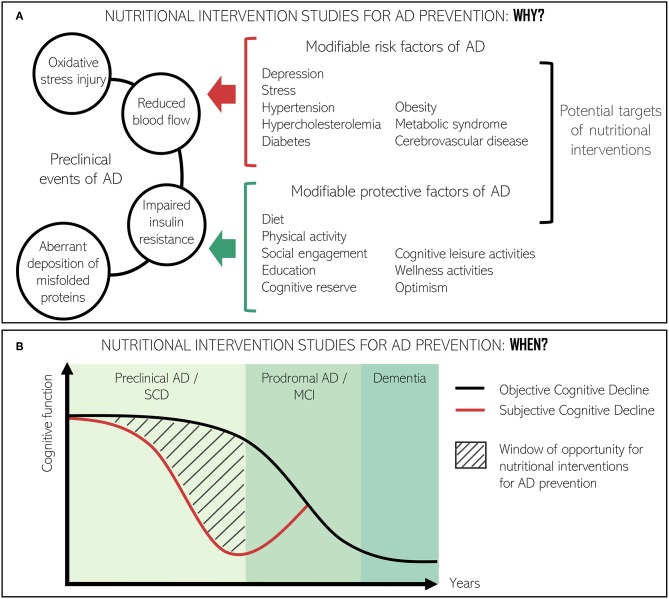
Conceptual framework for the relevance of nutritional interventions directed at improving cognitive function or slowing down cognitive decline at preclinical stages of AD. **(A)** Role of nutritional interventions in the interaction between preclinical events of AD and modifiable risk and protective factors of AD. **(B)** Window of opportunity to study the effect of nutrition interventions in the AD continuum. Adapted from Ávila-Villanueva and Fernández-Blázquez (42).

Preclinical pathological events of dementia include the exacerbation of certain age-related processes, such as reduced blood flow due to atherosclerosis, impaired insulin resistance, oxidative stress injury, and widespread, chronic, low-grade inflammation (41). In the preclinical stages of AD, there is also an aberrant deposition of misfolded proteins, called extracellular amyloid β plaques (Aβ) and intracellular tau-based neurofibrillary tangles, which contribute to the pathogenesis of brain atrophic lesions and, hence, cognitive deterioration (Figure 2A) (40, 43).

Preclinical events are grouped under the term subjective cognitive decline (SCD). SCD is considered the precursor to mild cognitive impairment (MCI) and potentially the earliest clinical sign of AD (44). SCD implies the subjective expression and experience of daily forgetfulness whilst performance on cognitive tests is normal (45, 46). Not every individual with characteristics suggestive of SCD will necessarily progress to AD. Nevertheless, recognition of the condition has critical clinical implications not only for patients and their physicians, but also for the identification of new target populations for clinical trials in the prevention of AD (47) (Figure 2B). On the other hand, MCI is considered a prodromal stage of AD although it does not strictly imply progression to clinically defined dementia (48). Use of the MCI category has been a matter of debate (9, 49) since it is irregularly defined and some subtypes are more likely to progress to dementia than others (50).

The mechanisms underlying the progress from preclinical AD to the symptomatic or prodromal stage of MCI, and ultimately AD dementia, are not clearly defined (51). In fact, in the 90+ Autopsy Study, nearly 50% of individuals aged 90 years or older with dementia did not have enough neuropathology in their brain to explain their cognitive symptoms (52). In contrast, around 30% of those older adults without dementia or cognitive impairment presented intermediate or high levels of AD pathology (53). Therefore, AD risk factors might contribute to the neuropathological process through mechanisms other than amyloid or tau (8). On the other hand, certain compensatory factors (e.g., maintenance of cardiovascular health, high educational level, social engagement) might capacitate individuals to tolerate considerable amounts of AD pathology without suffering a clear dementia syndrome, even in carriers of susceptibility alleles of genes such as apolipoprotein E (APOE) and the ε4 allele (19, 54).

Large individual differences in rates of age-related cognitive decline exist (55, 56), reflecting the complex interactions between cognitive performance and environmental and genetic factors (57). Evidence from human neuroimaging studies advocate that three mechanisms may partially mediate these differences: reserve, maintenance and compensation (57). Reserve is defined as the accumulation of neuronal resources during the lifespan (e.g., white matter quality of a fiber tract, functional connectivity), which help to mitigate neuronal decline caused by aging or in age-related diseases (57). Maintenance is defined as the preservation of brain resources via constant recovery and repair. Its efficacy depends on the magnitude of decline and the ability to repair (57). Finally, compensation represents the deployment of brain resources in response to task demands (57). Reserve, maintenance and compensation may act at the genetic, cellular and systems levels and can be influenced by several factors (57–67). Table 1 summarizes potential determinants contributing to cognitive reserve or cognition maintenance. These mechanisms can work simultaneously and interact between them. As exemplified by Cabeza et al. (57), education could augment cognitive reserve by increasing synaptic density, but this could only ameliorate cognitive decline if new synapses are well-maintained. Likewise, it is also necessary to deploy these resources during task performance, that is, to engage in compensation. These factors and its related mechanisms could explain the observed inter-individual variability in cognition, contributing to the degree of response to an intervention. For this reason, wherever possible, all these determinants should be measured when performing a clinical study.

**Table 1:** Examples of potential determinants of the inter-individual variability in cognitive decline.

**Determinant**	**Mechanisms**	**Example**
Gender Age Education Intelligence coefficient Development of expertise in a particular domain through training Bilingualism Socioeconomic factors	Reserve	Education improves neuronal resources during childhood and adulthood and ameliorate age-related cognitive decline in later adulthood. This beneficial effect may be partly mediated by the effects of education on health, stress, lifestyle and profession.
Occupational attainment Cognitive and social engagement Physical activity Diet Alcohol abuse Comorbidities Smoking Stress Genetic factors	Maintenance	Regular physical activity and exercise promotes neurogenesis and beneficial vascular changes which enhance brain maintenance.

The progress of AD from healthy aging to SCD, MCI and Alzheimer's dementia is an example of poor brain maintenance and it is modulated by reserve and compensation (57, 60, 68, 69). Adults with higher scores on reserve proxies (e.g., education or cognitive engagement) have less Aβ plaques, an accepted biomarker of AD (67, 70). Also, in adults with Aβ biomarkers, higher scores on reserve proxies are associated with a lower risk of progression from normal cognition to the onset of clinical symptoms (71).

In the absence of disease course-modifying treatments for AD, prevention is an emerging approach. A large number of modifiable AD risk factors have been identified in observational studies, ranging from environmental ones (e.g., air pollution) (72, 73) to lifestyle protective factors (e.g., physical activity and the Mediterranean diet) (74–78). Diet-related disorders (e.g., diabetes mellitus, obesity, metabolic syndrome, hypertension, and hypercholesterolemia) represent a considerable fraction of such modifiable risk factors (8). Therefore, nutrition interventions have the potential to reduce the incidence, delay the onset, and slow down the progression and severity of dementia in a cost-effective manner.

### Role of Phenolic Compounds, n-3 PUFAs and Vitamins in Cognitive Function

It is well-known nowadays that certain nutrients such as phenolic compounds, omega-3 polyunsaturated fatty acids (n-3 PUFAs), and vitamins, play a key role in the aging brain, possibly leading to better cognitive and motor capacity (79). These compounds are potent antioxidants and anti-inflammatory agents and are directly involved in signaling pathways that support brain plasticity (79).

Phenolic compounds are the secondary metabolites of plants found in a wide range of foods, particularly in red wine, olive oil, green tea, and blueberries (80). In both *in vitro* and *in vivo* models, phenolic compounds directly regulate amyloidosis, neuroinflammation, and tau aggregation (80–84). Several clinical trials have observed the positive cognitive effects of phenolic supplementation in cognitively healthy older adults (85–91) and those presenting mild to moderate dementia (92–97). Other trials, however, have failed to demonstrate these beneficial effects in the elderly (98–103). The poor bioavailability of phenolic compounds, difficulties in crossing the blood-brain barrier, the heterogeneity of the phenol dosage, and quality of the study design are several explanations for the lack of expected beneficial outcomes from clinical trials (10, 104, 105). Besides, inter-individual differences in the metabolism and bioavailability of these compounds can lead to different outcomes. For example, it is known that microbiota plays an important role in the gut bioactivation of certain phenolic compounds (106). Evidence is scarce, but some studies have associated specific microbiota composition with distinct enterolignan production (107) and with different clinical response to capsaicin supplementation (108).

n-3 PUFAs are structural components of cell membranes that act by displacing cholesterol from the cell membranes. Their function is crucial in maintaining cell integrity and achieving proper cognitive functioning (109). Among the n-3 PUFAs, particular attention has been focused on eicosapentaenoic acid and docosahexaenoic acid from oily fish, and its precursor alpha-linoleic acid from nuts. These n-3 PUFAs cannot be efficiently synthesized by human enzymes and are considered semi-essentials, relying almost exclusively on dietary intake (105). In AD animal models, long-term n-3 PUFAs supplementation has been shown to improve cognition and reduce the amount of deposited beta-amyloid (110, 111). Recently, the European Food Safety Authority has approved a claim on “docosahexaenoic acid and improvement of memory function” (112). However, inconsistent results have been obtained from clinical studies with fish oil supplementation in healthy older adults (113–118) and in patients presenting mild to moderate AD (119–123). One meta-analysis of ten RCTs suggested a protective effect of n-3 PUFAs within specific cognitive domains in patients with milder forms of cognitive impairment, but no effect was observed in healthy or AD subjects (124). In addition, a recent Cochrane review has concluded that n-3 PUFA supplementation cannot affect AD progression when the disease is already established (125). Future RCTs need to clearly define the optimal n-3 PUFA status for the aging brain, and the specific population that might benefit the most from n-3 PUFA supplements (79). The n-3 long chain PUFA biosynthetic pathway presents high inter-individual variability on their efficiency and is subjected to a high genetic diversity. This variability can explain inter-individual differences in the fate of n-3 supplementation and the n-3 daily requirements. As an example, fatty acid desaturase (FADS) regulates a key step on PUFA biosynthetic pathway. Polymorphisms in this enzyme trigger different plasmatic n-3 PUFA proportions in response to an alpha-linoleic acid enriched diet (126).

The role of vitamins on cognitive health has also been extensively studied although there is still a lack of clear conclusions (127). Optimal vitamin status is essential for the correct brain development and function. Deficiencies in several classes of vitamins have been associated with cognitive impairment and higher risk of dementia (128). In the case of vitamin D, the Endocrine Society recommends keeping vitamin D3 concentrations above 75 nmol/L (129). Several intervention studies have addressed the effects of vitamin D3 supplementation on cognition (130–133) with limited positive results (133). Among the vitamin B group, B12, B9 (folic acid), and B6 are involved in homocysteine metabolism, preventing the homocysteine-stimulation of oxidative stress (134) and their deficiency has been associated with cognitive impairment (135). Moreover, some promising results have supported vitamin B supplementation with respect to cognitive function (136), although there is as yet not enough evidence from RCTs to sustain its beneficial effect (137). Concerning vitamin C, MCI and AD patients present lower levels of ascorbic acid in plasma (138, 139). Long-term supplementation with vitamin C, however, has not been shown to be protective against developing AD (140). On the other hand, the supplementation of alpha-tocopherol, the most abundant class of vitamin E in the diet, and which plays a critical role in the protection of cell membranes from peroxidation, (141) has revealed some clinical evidence of potential benefit (142). Nevertheless, more trials are needed to confirm such results (143). Finally, β-carotene (provitamin A) presents anti-amyloid properties *in vitro*, but its effects in the aging population needs to be further studied (144).

Several clinical trials have addressed the question of whether multi-nutrient supplementation could have an impact on cognitive function. Fortasyn Connect is a patented mix of nutrients that act as precursors and cofactors of neuronal membrane formation. In animal models, it has been described as increasing synaptic function and formation (145, 146). However, a 24-month, double-blind RCT testing the efficacy of Souvenaid, a medical drink that contains Fortasyn Connect, has not been capable of showing an improve in cognitive function in individuals with prodromal AD (147). Nevertheless, in the same RCT with less impaired subjects, promising results were observed with respect to cognitive performance, which further support interest in investigating nutritional interventions in pre-clinical stages of dementia. Other nutraceutical formulations of phenolic compounds, vitamins, and n-3 PUFA have not shown a positive impact on global cognitive function in MCI, AD patients, and cognitively healthy older adults (148–150).

Special precaution should be exercised when analyzing the effects of food additives or supplements on a subject's cognition. Creating false expectations among consumers, as well as unexpected safety problems, should be avoided since the marketing of such products requires very little efficacy and safety evidence (19). Nutrient supplementations in nutraceutical doses may have an opposite effect compared to the nutrients provided in dietary doses. For example, a Cochrane review concluded that the antioxidants vitamin E and vitamin A, taken as supplements, increased mortality in healthy participants and patients with various diseases (151). Therefore, caution should be taken before recommending high-dose supplements of nutrients.

### Mediterranean Diet and Cognitive Function

Over the past years, the main focus of research in nutrition has shifted from the study of individual nutrients or single foods to the study of complete dietary patterns which better reflect the whole diet of a population (152). The Mediterranean diet (MeDiet) is a healthy dietary pattern characterized by a high intake of vegetables, fruit, legumes, olive oil, fish, cereals, and nuts, along with moderate red wine consumption during meals (153, 154). These components make MeDiet rich in phenolic compounds, n-3 PUFA, and vitamins that, in conjunction, may contribute to a better neurovascular health, and reduced oxidative stress and chronic inflammation (7, 155). Multiple reviews and meta-analyses of observational studies have underlined the strong association between the traditional MeDiet and improved cognitive function, and reduced risk of cognitive decline and dementia, delayed AD onset, and lower mortality in AD patients (7, 156–158). Further valuable evidence of the effects of MeDiet on cognition comes from the PREDIMED study, a RCT designed to test the cardiovascular effects of MeDiet among older adults at high cardiovascular risk (159). Volunteers who followed a MeDiet enriched with extra virgin olive oil or nuts for up to seven years (median of 4.8 years) were compared with a control group who followed a low-fat diet. Those adhering to a MeDiet for more than 4 years presented lower incidence of stroke (159) and an improved cognitive function (160, 161). Several mechanisms could explain the positive effects of MeDiet on cognitive function. Basically, a reduction of the risk factors that have independently been associated with increased risk of dementia (162).

Due to differences in food production, food availability, and cultural habits, a strict MeDiet cannot be extrapolated to all non-Mediterranean countries. Therefore, besides MeDiet, alternative healthy-dietary patterns have also been associated with better cognitive function and reduced risk of cognitive impairment. The blood pressure lowering DASH diet (Dietary Approach to Systolic Hypertension) was targeted exclusively to lower blood pressure in hypertensive and pre-hypertensive patients. Long-term adherence to the DASH diet (6 years) promoted the maintenance of global cognition and verbal memory in older adults (163). On the other hand, the MIND diet score (Mediterranean-DASH diet Intervention for Neurodegeneration Delay) represents a modified version of MeDiet that captures additional foods and nutrients of DASH diet (e.g., berries or green leafy vegetables) (164). In adjusted models, adherence to the MIND diet slowed cognitive decline (165) and decreased AD risk (166). Finally, the Nordic diet, followed in Scandinavian countries, is a diet rich in phenolic compounds, unsaturated fatty acids, and whole grain products. An observational study concluded that adherence to the Nordic diet was associated with a better cognitive performance in older adults with normal cognition (167).

### Multidomain Lifestyle Interventions

Multidomain lifestyle interventions deal with multiple modifiable risk factors of dementia simultaneously, including nutrition, cognitive training, social support, physical activity, and the management of the vascular risk factors (Table 2) (177). These interventions are mainly justified by the heterogeneous etiology and pathogenesis of dementia, including the numerous pathways and domains involved in cognitive performance that may be affected in the brain degeneration process (9, 177). The promotion of adequate brain stimulation through cognitive training and social support is thought to be useful to build new neuronal pathways and retain the remaining ones (177). This, in turn, increases cognitive reserve, defined as the capacity to perform cognitive tasks properly despite neuropathological damage to the brain (178, 179). Another key point of multidomain interventions is caring for nutritional status, as has been previously mentioned, the lack of specific nutrients affects brain degeneration and some dietary patterns are associated with better cognitive performance (177). Finally, adequate blood flow is important in preventing cognitive decline and also facilitates the efficient Aβ clearance from the brain (180). Therefore, physical activity or medications that promote cerebrovascular health are frequently included in multidomain interventions, as they may be helpful even when dementia is already established (177, 181, 182).

**Table 2:** RCTs of multidomain lifestyle interventions for prevention of cognitive impairment, Alzheimer's disease, and dementia.

**Study (Ref) location**	***N***	**Inclusion criteria**	**Multidomain intervention**	**Control**	**Duration**	**Primary outcome**	**Results *(if available)***
FINGER (168) Finland	1,260	Cognitive performance at mean level or slightly lower than expected for age 60–75 (CAIDE dementia risk score ≥6) 60–77 years	nutritional guidance physical exercise cognitive training and social activity management of metabolic and vascular risk factors	General health advice	2 years + 5 years follow up	Change in cognitive function (NTB)	Significantly positive effects
preDIVA (169) Netherlands	3,526	Unselected population of older people without dementia in general practices 70–78 years	nutritional advice physical activity advice vascular care and medical treatment of risk factors	Usual care	6 years	Cumulative incidence of dementia and disability score (ALDS)	No significant effects
MAPT (170) France	1,680	Spontaneous memory complaint (MMSE >24), with frailty (limitation in one instrumental activity of daily living and slow walking speed) ≥70 years	nutritional advice physical activity advice cognitive training vascular care and/or 800 mg DHA/day	Placebo	3 years + 2 years follow up	Change in cognitive function (G and B)	No significant effects
Lam et al. (171) Hong Kong	555	MCI ≥ 60 years	physical exercise cognitive activity	Social activity or only cognitive activity or only physical exercise	1 year	Change in cognitive function (CDR-SOB)	No significant effects
HATICE (172) Netherlands, Finland, France	2,600	healthy cognitive status (MMSE ≥24), with cardiovascular risk factors ≥65 years	Interactive internet platform that stimulates self-management of vascular and life-style related risk factors, with remote support	Static internet platform with basic health info	1.5 years	Composite score based on the average z-score of the difference between baseline and 18 m follow up values of BP, LDL, and BMI	N/A
SYNERGIC (173) Canada	200	MCI 60–85 years	exercise cognitive training vitamin D	BAT, control cognitive training, placebo D	20 weeks + 6 month follow-up	Change in cognitive function (ADAS-Cog 13 and plus)	N/A
LIILAC (174) Australia	148	Healthy cognitive status (MMSE >24) 60–90 years	MeDiet exercise	Usual care or only MeDiet or only exercise	6 months	Change in cognitive function (SUCCAB)	N/A
Daly et al. (175) Australia	152	Healthy cognitive status (SPMSQ ≤ 2) ≥65 years	progressive resistance training lean red meat vitamin D	Control resistance training, advice to consume carbohydrates and vitamin D	6 months + 6 month follow up	Change in cognitive function (CogState Battery)	N/A
Rovner et al. (176) USA	200	African Americans with MCI ≥ 65 years	Behavior activation therapy to help subjects develop strategies to maintain cognitive, social and physical activities	Supportive therapy	2 years	Change in episodic memory (HVLT-R)	N/A

*FINGER, Finnish Geriatric Intervention Study; MAPT, Multidomain Alzheimer Prevention Study to Prevent Cognitive Impairment and Disability; preDIVA, Prevention of Dementia by Intensive Vascular Care; HATICE, Healthy Aging Through Internet Counseling in the Elderly; SYNERGIC, Synchronizing Exercises, Remedies in Gait and Cognition; LIILAC, Lifestyle Intervention in Independent Living Aged Care; ADAS-Cog, Alzheimer Disease Assessment Scale Cognitive 13 and the plus modality; ALDS, Academic Medical Center Linear Disability Score; BAT, balance and toning exercise; BMI, body mass index; BP, systolic blood pressure; CDR-SOB, Clinical Dementia Rating sum of boxes; CogState Battery, CogState Brief Battery computerized tests; G and B, Grober and Buschke; HVLT-R, Hopkins Verbal Learning Test-Revised; LDL, low-density-lipoprotein; MCI, mild cognitive impairment; MMSE, Mini-Mental State Examination; N/A, not available; NTB, Neuropsychological Test Battery; SPMSQ, Short Portable Mental Status Questionnaire; SUCCAB, Swinburne University Computerized Cognitive Assessment Battery*.

Up to the present, several studies have employed a multidomain lifestyle approach to prevent cognitive decline, dementia, and AD. (Table 2) (168–170, 172–174). In the Finnish Geriatric Intervention Study to Prevent Cognitive Impairment and Disability (FINGER), 1,260 older adults at risk of cardiovascular disease and dementia were included. Participants were randomized to receive a regular health advice or a 2-year lifestyle multidomain intervention which included nutritional guidance, group and individual physical activity, cognitive training, and intensive monitoring of vascular and metabolic risk factors (183). The primary outcome of cognitive performance was measured using the Neuropsychological Test Battery (NTB) total score (168). NTB total score was statistically higher in the intervention group, hence, the multidomain intervention was able to improve global cognition (168). One of the key factors of the FINGER success was thought to be the combination of group and individualized activities, which increased both personal and within-group motivation and enhanced lifestyle changes (6).

Nonetheless, the two other large, long-term, multidomain, lifestyle-based studies failed to obtain successful results. The Prevention of Dementia by Intensive Vascular Care Trial (PreDIVA) targeted vascular risk factors to prevent the incidence of dementia in 3500 cognitively healthy older adults (169). Despite no statistically significant effects of the intervention in the whole group (hazard ratio was 0.92, 95% CI 0.71–1.19; *p* = 0.54), certain benefits on dementia incidence were found in at-risk subgroups (169). On the other hand, the Multidomain Alzheimer Preventive Trial (MAPT) included a 3-year n-3 PUFA supplementation (DHA+ EPA) combined with a lifestyle intervention in order to prevent cognitive decline in 1,680 older adults with subjective memory complaints (170). Likewise, only the individuals presenting a higher degree of cognitive impairment benefited from the intervention (170). Findings from these studies suggest that interventions should be specific for risk profiles (6, 184, 185).

## Personalized Nutrition

Personalized nutrition is based on the idea that individuals respond differently to dietary components as a result of the interplay between environmental, social, metabolic, and genetic factors. Targeting these variations can have an impact on final health status. Personalized nutrition provides tailored advice adapted to the individual's unique characteristics with the aim of promoting a sustainable change beneficial for health. The basis of personalization has not yet been established. It can be established with biological data such as genotype/phenotype, or with a more behavioral approach, including preferences and socioeconomic determinants such as gender, cultural aspects, and access to food (33). Some authors have suggested “a shared decision-making approach” as a tool for personalizing dietary advices and thus increasing acceptance and adherence (33).

The study conducted by Zeevi et al. (186) represents a proof-of-concept for the feasibility of personalized nutrition. They developed a machine-learning algorithm integrating blood parameters, anthropometric measures, gut microbiome data, and dietary and lifestyle information, in order to predict an individual's postprandial glycemic response. They validated the algorithm in an independent cohort and demonstrated that a dietary intervention based on their prediction lowered postprandial responses. Another study worth highlighting is Food4Me which is, to date, the largest RCT in personalized nutrition (187). It demonstrated that personalized nutrition was more effective than standard population advice in relation to dietary behavior. Personalization was based on weight, physical activity, and dietary intake. Interestingly, the inclusion of phenotypic/genotypic data to define the recommendation did not produce additional benefits (187–189). Experts in the field of nutritional genetics and genomics agree that more research is needed to implement such approaches within the scope of evidence-based nutrition (190, 191).

Nevertheless, no personalized nutritional study has been carried out with the aim of improving cognitive function, slowing cognitive decline, or reducing dementia incidence. The implementation of personalized nutrition in clinical trials faces a considerable number of practical, logistical, and financial challenges. As stated by the epidemiologist Geoffrey Rose, personal lifestyle is socially conditioned, which means that individuals are unlikely to eat very differently from the rest of their families and social circle. Current personalized clinical and preventive nutritional approaches are more designed to help individuals rather than entire populations. The challenge for research will be to define efficient personalization methods for increasing the impact of lifestyle interventions on the global burden of dementia and cognitive decline, as well as reducing health disparities when implemented on a large scale (33).

## Methodological Challenges of Nutrition Trials for Dementia Prevention

Despite the increasing interest in the possible relationship between nutrition and cognitive health, evidence indicating the preventive effectiveness of nutritional strategies remains scarce. Encouraging results from *in vitro*, animal, and epidemiological studies have not been automatically translated into successful results in RCTs. This inconsistency has been attributed to methodological difficulties in performing nutrition preventive RCTs, particularly against cognitive disorders and, more generally, age-related pathological conditions (Table 3) (192, 193).

**Table 3:** Challenges of traditional clinical trials testing the efficacy of nutritional interventions for dementia prevention.

**Main challenges**	**Risks or consequences**
**Study Design**	
Blinding	Control group contamination
Adherence to the intervention	Poor sustainability of the proposed intervention
Dropout rates	Decreased statistical power
	Confounding and selection biases post-baseline
**Dietary assessment**	Misreporting and underreporting concerns due to memory difficulties of participants experiencing cognitive decline Participant and researcher burden
**Cognition as an outcome**	
Short interventions	Incapacity to assess effects on primary outcomes (e.g., dementia incidence) Limited clinical relevance of minor cognitive changes
Cognitive assessment tools	Poor sensitivity of cognitive tests to detect subtle changes in cognition
**Target population**	
Heterogeneity of target population Strict inclusion/exclusion criteria	Inconclusive results Healthy subsample of the target population Potential effects of disclosing to participants a high risk of developing AD

### Study Design

Conventional parallel-group RCTs with primary clinical endpoints represent the peak of the study design hierarchy due to their ability to infer causality between an exposure and an outcome. They represent the main basis for medical guidelines and health policies (194). Double blind RCTs minimize confounding and selection biases at baseline through randomizing the allocation of exposure (195), which is a major challenge in observational studies. Therefore, through RCTs it is theoretically possible to carry out a pure comparison between intervention and control arms (195). Although they are a fundamental design in drug efficacy study paradigms, traditional RCTs may not be easily extrapolated to the nutritional field (196).

One major drawback in nutritional clinical studies is that blinding a dietary intervention is not always possible. As a result, the adherence and effect of the intervention can be affected by the possible knowledge of the treatment assignment (195–197). Moreover, dropout rates are usually higher in RCT nutritional interventions compared to drug trials, particularly if the intervention is very demanding or is carried out during a long time period (196). This consequently reduces statistical power and, if a selective dropout occurs between groups, it can lead to confounding and selection biases post-baseline (196). Furthermore, adherence to the assigned intervention is difficult to achieve (and assess) in nutritional trials of long duration or when the assigned intervention substantially differs from the participant's usual diet (194, 196, 197). Such constraints not only affect the study results, but also call into question the sustainability of dietary interventions.

For ethical and methodological reasons, the control group of nutritional RCTs is not typically placebo-controlled, but instead it is often a low-dose group, which can cause poor contrasts among groups (196). In addition, before entering the study, all participants are informed about the potential effects of the intervention. Therefore, there is always the possibility that control subjects look for dietary components that mimic the intervention (195). All these limitations regarding the choice of the control group can mask the true effect of a nutritional intervention (196).

### Traditional Dietary Assessment

Accurate dietary assessment is one of the main challenges researchers face when performing nutritional studies. The complexity of measuring diet is increased by the constant introduction of new products and trends which have an impact on consumers' decisions (198). This issue has worse perspectives when dealing with older people. The elderly make up a very heterogeneous group, with a wide range of ages, health and physical status, cognitive situation, and socioeconomic level. Aging is associated with several changes that can affect directly or indirectly dietary intake and nutritional status. There is a loss of smell and taste, difficulties in chewing and swallowing, and changes in living conditions that can all have an impact on dietary intake (199).

Food frequency questionnaires (FFQ), food diaries, and 24-h dietary recalls are the three main traditional methods of dietary assessment. The 7-day weighed food record is the technique that can most capture details and variation in the diet compared to the FFQ or 24-h dietary recall (200). However, prolonged recording of 7-day weighed food records has been linked to mis- and under-reporting as it may be too demanding for the participant, and changes in normal behavior and dietary habits (201). The goal of dietary assessment is to achieve a balance between the collection of reliable and accurate data, and the burden for the patient and researcher. In order to do so, the National Cancer Institute has standardized an automated web-based self-administered 24-h recall tool, ASA24. A study has shown that self-administration of at least 3 ASA24 on separate days provided equivalent results to a 4-day food diary. This represented a cost-benefit improvement in terms of data collection and management (202). Nevertheless, methods based on self-reporting data are prone to substantial misreporting. They challenge the participant's memory and capacity to estimate food content and portion size (203) which become even less reliable when participants present cognitive decline. The tendency to under-report in dietary assessment has been not only associated with body weight and BMI, but also with gender, sociodemographics, lifestyle, education, and diet characteristics. It has been reported that the elderly tend to under-report in 24-h dietary recalls due memory difficulties and cognitive loss (203). Traditional dietary assessment methods are currently being re-defined with the development of new technological tools such as m-health, machine learning, and food image recognition models. Simultaneously, the rise of the ‘omics techniques has enabled the development of new biomarkers capable of assessing dietary exposure.

### Cognitive Outcomes

The choice of the method used to assess cognitive performance is crucial to obtain successful results. Primary cognitive outcomes should be unambiguous and clinically relevant, such as reducing the incidence of dementia or delaying its onset. Nevertheless, such objectives require large sample sizes with long-term follow-ups when intervening at the age of 60–70 or earlier (192). Short-term preventive interventions are usually unable to evaluate the intervention effects on “hard outcomes.” Instead, they measure cognitive function by means of a composite score that includes a battery of validated cognitive tests administered at discrete intervals. Although cognitive change is an indicator of progression across the disease continuum, the clinical importance of the observed cognitive changes remains unclear (193). In addition, the sensitivity of cognitive tests to detect subtle individual cognitive changes due to nutritional interventions in the earliest stages of dementia has been questioned. There is no consensus about the gold standard tool to measure cognitive decline (204). Experts recommend including functional measures, health-related quality of life, health care utilization, and institutionalization as outcomes for dementia preventive trials.

### Target Population

Defining the ideal target population is challenging due to the nature of the development and progression of dementia, as there is a delay between risk exposure, disease onset, and clinical manifestation (192). The optimal age for preventive trials is still unknown: Acting prematurely in the natural history of the disease would require a too long a follow-up to be realistic, while intervening too late might result in too little efficacy (192). The current target population groups for preventive trials are usually cognitively intact individuals or those presenting SCD/MCI.

Such groups are largely heterogeneous and the probability of short-term changes in cognition is low. Consequently, there is the need to increase sample size, length of intervention, and, as a consequence, human and financial resources (49). In order to homogenize study samples, the presence of several biomarkers of cognitive decline is usually reflected in the inclusion criteria, while having comorbidities or poor levels of education are part of the exclusion criteria. However, the disclosure of biomarker positivity among study participants carries potential risks for the individual, including fear or anxiety about the future, which can influence the subsequent neuropsychological testing or perception of clinical decline (205). In addition, compared to pharmacological trials, the use of an extensive inclusion/exclusion criteria is not particularly relevant in nutrition interventions, as contraindications are not a primary concern (49). Strict inclusion/exclusion criteria merely lead to a healthier subsample of the target population (49).

Given the limitations of traditional RCTs, many researchers are expressing the need for novel patient-centered experimental designs such as N-of-1 trials, in order to achieve individualized, targeted treatments, and recommendations for clinical care (8, 195, 206, 207).

## N-of-1 Clinical Trials

### Rationale

Dementia and AD are characterized by a wide heterogeneity in terms of risk factors and clinical manifestation and progression. Such heterogeneity has been recognized as a critical issue in the current approach to implement preventive strategies and develop new therapies (208). For example, genetics account for more than 50% of the phenotypic variance of late-onset AD (209), and there are gender differences in the phenotype and progression of AD (208). Nutritional interventions also encompass large heterogeneity among individuals because environmental, social, metabolic, and genetic factors influence response to dietary interventions. According to the principle of equifinality, the earlier the disease process is, the higher the inter-individual variability. Therefore, it is essential to explore the heterogeneity involved in nutritional interventions directed toward improving cognitive function in order to achieve the final goal of being able to make individual-based treatment decisions.

Nevertheless, evidence from traditional clinical trials is probabilistic and there is no certainty of individual benefit (33). This is because parallel-group RCTs estimate the average treatment effect of the studied intervention, since randomization theoretically ensures overall comparability among treatment groups. The absence of differences in baseline characteristics among study groups does not exclude the possibility that some individuals respond or benefit to a greater or lesser degree than the reported average. This is referred to as heterogeneity of treatment effects (HTE) (210) which can be attributed to treatment, patient, or environmental factors, and it is usually presented as considerable variations around group means. Examining the HTE is central to patient-centered outcome research and for informing personalized treatment decisions (211).

The most common approaches to exploring HTE in clinical trials are subgroup analyses, the stratification of patients according to risk profiles, and meta-analysis (18, 210). Subgroup analyses are typically performed to detect characteristics among the study population that are associated with a greater benefit from the intervention, no benefit, and even with harm (212). Nevertheless, subgroup analyses may not be able to detect clinically significant differences in treatment effects among participants when multiple factors determine the risk (213). One alternative is to consider risk-stratified analyses. Participant stratification, using a multivariate risk model, makes it possible to compare the treatment effect across a broader range of risk factors or patient attributes than a conventional one-variable-at-a-time subgroup analysis (213). Nevertheless, such stratification is not always feasible in RCTs that target dementia or AD, since the available biomarkers only detect the presence of the disease, but not its onset and progression. In addition, although some risk scores exist (214, 215), there is a limited understanding of the interplay among individual dementia risk factors during the life course, and how they affect the pathophysiology of the disease and impact on the brain health (216). Finally, regarding meta-analysis, it is difficult to pool multiple RCTs due to differences in the diagnostic procedure and criteria for dementia and AD, as well as variations in the measures used, anticipated outcomes, and study design. N-of-1 trials can overcome the difficulties of parallel group RCTs in determining HTE as they focus on individuals in order to establish their optimal treatment.

### Design and Indications

The design of an N-of-1 clinical trial is determined by the research question to be answered, in other words, by the nature of the intervention and the characteristics of the outcome to be measured. In the case of nutritional studies, the intervention can range from a single-compound intervention to a complete change of dietary pattern or a multidomain intervention. The first group of studies could adopt a more pharmacological N-of-1 approach whereas the second group of interventions would require a more complex study design including behavioral changes, learning, and habituation periods.

#### Traditional N-of-1 Approach

To perform a traditional N-of-1 design, several assumptions must be met. First, it is indicated when there is uncertainty regarding the comparative effectiveness of an individual's treatment, and when significant individual differences in intervention response are expected (20, 217). Second, the study disease must be chronic, stable, or slowly progressing, with evaluable symptoms or valid biomarkers to allow monitoring during the course of the study (20, 217). Finally, interventions must be reversible, have rapid efficacy and minimal carryover, tolerate wash-out periods (if necessary), and multiple-crossovers (20, 217).

In the case of a single-compound, nutritional intervention, a traditional N-of-1 approach can be followed as in drug efficacy N-of-1 studies. The participant undergoes two or more nutritional interventions in a random order (e.g., A-B-A, A-B-A-B, or A-B-A-C-A-D), and this process may be repeated several times (in series of N-of-1 trials) (17). Therefore, the individual treatment effects can be re-estimated after each intervention period. The number of time-series conducted can be pre-specified or adapted in order to stop the trial when there is reasonable statistical certainty to identify the most effective treatment for the participant (18). The aggregation of data from series of N-of-1 trials allows the possibility of controlling for random or patient fixed effects, as well as covariates, sequence or center effects (18). Ultimately, this design enables the identification of individuals who share a response profile tackling the similarities between them (206, 218). Thus, this single-person, multiple crossover, randomized trial design is able to provide direct and objective evidence about the value of a particular single-compound or nutritional intervention for the patient, and also for the general population (217).

An example of this traditional N-of-1 approach is the recently started Personalized Research on Diet in Ulcerative Colitis and Crohn's Disease (PRODUCE) trial (NCT03301311). Using series of N-of-1 trials, patients will cross-alternate between two different carbohydrate diets in order to determine their effects in reducing symptoms and inflammatory burden at both individual and population level.

#### Modified N-of-1 Design for Studying Cognitive Decline Preventive Interventions

In the field of dementia prevention, current evidence points towards multidomain interventions addressed to individuals at high risk. These types of interventions imply behavioral changes that are difficult to reverse, so it is hard to imagine the possibility of conducting multiple crossovers in a single person. In addition, cognitive outcomes are not susceptible to short-term changes and there are not currently sensitive biomarkers of disease progression.

**Figure 3:**
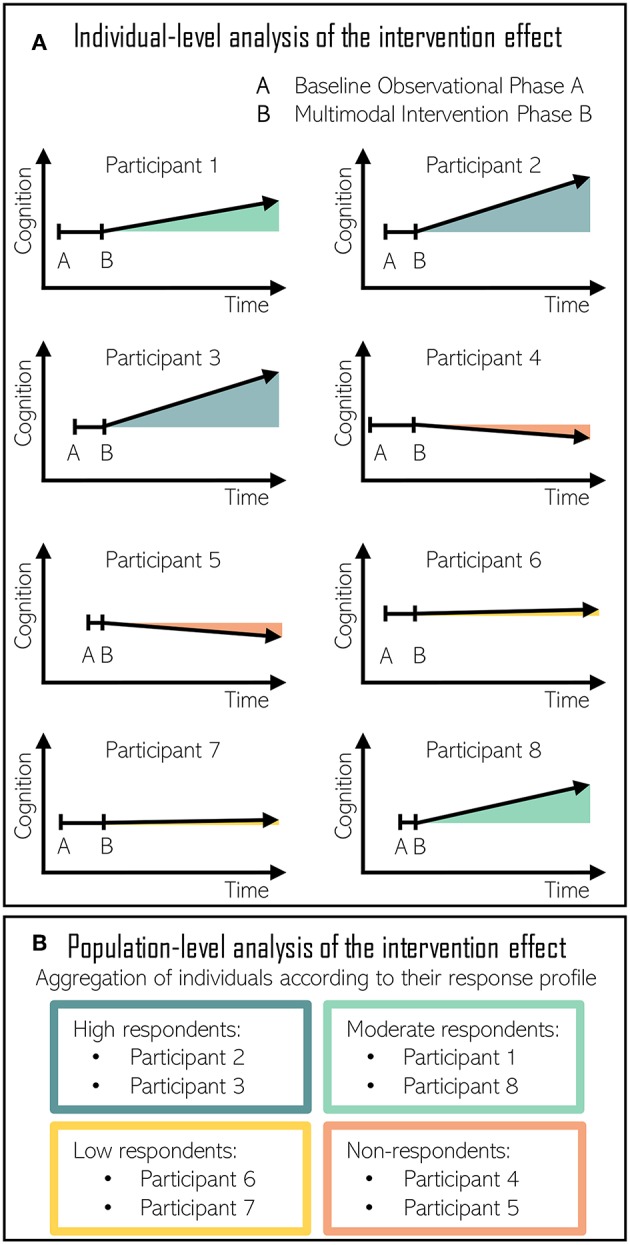
**(A)** Individual-level analysis of the intervention effect in N-of-1 nutritional studies directed at improving cognitive function. Baseline observational phase A duration is randomized among volunteers. Multimodal intervention phase B triggers a different response to each individual. **(B)** Population-level analysis of the intervention effect. Individuals are aggregated according to their response. High respondents and moderate respondents improved their cognitive performance following the intervention. Low respondents maintained cognitive performance with the intervention. Finally, cognitive decline progressed in non-respondents despite the intervention.

Our proposal is a modified N-of-1 approach, which would start with a “baseline phase A” where a participant would undergo through an extensive evaluation of his or her lifestyle habits, socioeconomic status, medical history, and laboratory and cognitive assessments (219). Baseline data should appear stable before the implementation of the intervention in order to ensure that cognitive function changes when, and only when, the multidomain intervention is introduced for all participants. During “intervention phase B,” researchers would repeatedly assess adherence to the targeted behavior and the evolution of participant's cognitive status, making use of the latest technological devices for recording continuous health data (Figure 3).

As the intervention effect estimation is based on comparisons between the intervention phase B and the baseline phase A, the estimation is susceptible to bias when events other than intervention cause shifts in the time-series (220). In these occasions, the assurance of the stability of the baseline period is crucial. Moreover, the use of a multiple baseline design can improve the accuracy of the baseline phase by raising awareness among researchers about the presence of potential threats to internal validity (220). In the case of AB study designs, the combination of several AB experiments in multiple subjects and the addition of multiple-baseline increases the power of the observations (221). In such cases, the length of the baseline period varies systematically and is randomized for each participant. As exemplified in Figure 3, participant 1 would start the multidomain intervention after 1 baseline month, another after 1.5 baseline months, then 2, and so on. The staggered introduction of the intervention allows for separation of intervention effects from those of maturation, experience, learning, and practice (220, 222). Consequently, multiple baseline designs are strongly recommended in single-case intervention studies when reversal designs are not feasible (221).

Concerning the statistical analysis, it is recommended to, first, perform a visual analysis (e.g., by means of a spaghetti plot) in order to determine whether there is a functional relation between the intervention and the outcome. The visual analysis includes the evaluation of the level (mean score of the data within a phase), the trend (the slope of the best-fitting straight line for the data within phase), and the variability (the fluctuation of the data around the mean) (222). If there is a potential functional relationship between intervention and outcome, a second step is to perform a quantitative analysis, firstly at the individual level and secondly at the population level (between-subjects). As there is not a consensus about which quantitative methods are more appropriate, some authors recommend to conduct a sensitivity analysis and to report multiple effect size estimators (222). If there is consistency across different effect size estimators, there is stronger evidence for the efficacy of the intervention (222).

Therefore, the aggregation of data from multiple N-of-1 trials studying the same lifestyle interventions could be used to explore trends in data that may reveal the characteristics of participants responding to the intervention, as well as confounding factors that could be incorporated into the analysis of the intervention effects and future trials (207). The meta-analysis of multiple N-of-1 trials would therefore take advantage of the continuous and intensive follow-up of participants. It could thus be possible to obtain sufficiently detailed information from each participant to achieve a high level of evidence of the intervention effects for both the individual and the population.

#### Second Step N-of-1 Studies

The proposed N-of-1 study approach could also be applied as a second step in the analysis of large concluded intervention studies (e.g., FINGER, preDIVA, or MAPT). This would allow the examination of the size of the intervention effect at an individual level. This approach would enable sub-group identification of treatment respondents and non-respondents and the classification of factors that could be influencing treatment response and treatment adherence. Furthermore, the sub-group data aggregation would allow the accurate analysis of biomarker trends and changes within the study to better understand the disease progression within sub-groups. Nevertheless, to do so, N-of-1 assumptions would need to be met before performing the analysis. Firstly, to have performed a continuous monitoring of the volunteers' treatment adherence and response to be able to detect and predict changes in treatment responses. Secondly, a complete characterization of the volunteers is needed to understand the heterogeneity of the obtained results. Lastly, the stability of the studied condition before treatment initiation should be ensured. In the case that stability cannot be warranted, causality could also be determined when changes are observed in a sub-group only in the treatment intervention but not in the control intervention. In this situation, the power associated to within-subject comparison would be lost. This second step N-of-1 analysis would provide valuable information to personalize treatment and to predict and prevent condition evolution. This would be an exploratory approach, and based on the acquired knowledge, it could be used as preliminary data to perform a confirming first step N-of-1 trial.

### Main Challenges of N-of-1 Trials

#### Lack of Control Group

In the proposed modified N-of-1 approach, there is no control intervention, just a baseline phase, which is used to determine the size of the effect. In our proposed N-of-1 study design, determination of treatment causality would require first, a stable baseline, and second, an observable change coinciding with the intervention. Causal determination would be reinforced by the replication of the results in further subjects (223). As previously stated, the multiple baseline design and the continuous follow-up of participants can improve the internal validity of the results. However, the lack of a control group makes it difficult to match non-specific effects of the intervention such as expectation or attention.

#### Monitoring Behavior Over Time

In N-of-1 trials it is necessary to measure behavior over time in order to obtain a representative picture of each participant's lifestyle habits. The monitoring process can itself produce changes in behavior due to the awareness of being monitored, which is known as participant reactivity. This can lead to inaccurate baseline data, jeopardizing the determination of the intervention effects (224). In addition, whilst future technology promises high quality objective dietary and cognitive monitoring, current approaches still need to overcome some challenges.

##### Continuous diet assessment

N-of-1 nutritional studies seek a continuous and intensive monitoring of the volunteers in order to examine dietary choices and assess the response to specific dietary components. At present, traditional diet assessment methods do not enable continuous assessment, technology is thus aiming to improve and adapt these tools. Technology-based approaches have great potential, reducing both patient and investigator burden, as well as decreasing economic and time investment (198, 225, 226). For instance, image-assisted dietary assessment tools have appeared in order to estimate energy and nutrient intake. Several pilot studies have demonstrated that images provide improved self-reported dietary intake by decreasing the unreported/misreported errors. However, food image recognition fails when there are hidden ingredients, and cooking methods and cultural aspects that change nutritional composition (227). Consequently, whilst image-assisted dietary assessments improve dietary error they still need to be supported by additional information. Likewise, special consideration needs to be given to the use of technology-based tools for dietary assessment in older adults as most of these approaches have been tested with younger people. The elderly may be reticent in the use of computer-based technology, encountering difficulties in its management, which may lead to unreliable diet information.

Dietary biomarkers have appeared as the optimal way to measure exposure to a certain food item or nutrient, they however, require equipment and expertise (198). Moreover, they may need invasive specimens such as blood or feces. Biomarkers can change their concentrations depending on individual characteristics including gender, absorption, metabolism, genetics, microbiota, and environment (228).

The development of high-throughput, technology-based –omics research, especially metabolomics, has appeared as a new tool to assess an overall picture of dietary intake. Food metabolomics can identify novel diet-related biomarkers and investigate the mechanism of action behind nutritional interventions through changes in metabolic pathways. The food metabolome is highly complex and variable, nevertheless, it provides a unique and rich source of information regarding an individual's diet (229). Inter-subject variation, and the short life of certain metabolites which may not represent usual intakes, need to be taken into consideration. A biomarker has to be sensitive to intake, food specific, and easy to measure. A single measurement of the metabolome is not sufficient to provide an overview of a long-term dietary pattern (228). Efforts have been made in order to identify the metabolomics fingerprint associated with different dietary patterns, going beyond single-food or single-nutrient biomarkers (229–231). These studies have been able to detect and identify new biomarkers associated with food patterns in free-living populations. Nevertheless, food metabolomics is still at an initial stage and represents a complement rather than a replacement of traditional diet assessment methods (232).

##### Continuous cognitive monitoring

Neuropsychological test batteries have high specificity and sensitivity for the detection of the current cognitive state. They do, however, require considerable resources and are quite time-consuming with respect to continuously monitoring alterations in the cognitive status during a clinical trial. New computerized cognitive tests are being developed for longitudinal cognitive monitoring (233). They have the potential to use random elements and alternate sequences to minimize learning effects, and they can adapt the testing difficulty to the baseline cognitive performance of each individual. In addition, traditional pencil-based or computerized self-reported cognitive tests can be combined with real-time sampling methods in a participant's natural environment (e.g., ecological momentary assessment–EMA) which could reduce retrospective reporting biases (224). Finally, web-based platforms of cognitive stimulation games can be used to longitudinally evaluate and monitor each participant's performance in specific cognitive domains.

#### Statistical Design and Analytic Considerations for N-of-1 Trials

The relevance and importance of N-of-1 trials is increasing and, as a result, there is an abundance of literature on statistical methodology (25, 206, 234). The importance of these trials is also reflected by the fact that the CONSORT (Consolidated Standards of Reporting Trials) statement has been extended for these type of studies (17). Several of the methodological challenges that N-of-1 trials encounter are the following:

- The determination of the number of observations needed for valid inference requires a careful planning of each trial. It will depend on the block design (i.e., the number and order of blocks per treatment) and prior knowledge of both the within and between-block variation. In addition, if several N-of-1 trials are planned with the purpose of pooling the results, between-patient variance will also play a key role to determine the number of parallel N-of-1 trials (20).
- Moreover, the computation of the number of measurements needed has to account for the fact that the data of N-of-1 trials have a time-series structure, which implies autocorrelation (31).
- Variables that can be recorded constantly by means of mobile applications, which provide individual time-series, can be analyzed with standard statistical techniques such as autoregressive integrated moving average (ARIMA) and autoregressive moving average (ARMA) models. However, the inclusion of the information of such time-series as a covariate in a regression model is not straightforward and various possibilities should be considered. For example, a time-series on physical activity could be summarized by means of the mean activity per day, the cumulative activity, or a moving average over several days.
- Pooling the results of several N-of-1 trials can be carried out in different ways and will depend on the data at hand. On one hand, the results from several trials could be jointly analyzed by means of linear mixed models or meta-analysis (25). However, there might be substantial differences among the patients of several N-of-1 trials and this data heterogeneity could complicate such an analysis. Another approach is to identify patients with similar response profiles and subsequently study what they have in common (206). For this purpose, graphical inspections of the resulting time series in the case of continuously recorded variables could be used.

## Conclusion

Advances in the field of dementia prevention require the integration of evidence from multiple study designs, technologies, and methodologies with complementary strengths and weaknesses (235). We propose N-of-1 or single person clinical trials to address some of the current shortcomings of dementia prevention trials. They can provide knowledge concerning individual differences in the response to dietary interventions, which may reveal insights of the mechanisms behind interventions. In addition, the longitudinal personal data acquisition will increase the value of each participant's contribution in the trial by improving the characterization of previously unmeasured confounders (236). N-of-1 trials also offer the potential to evaluate and predict intervention effects in populations that are typically excluded from clinical trials, such as the elderly with multiple comorbidities or individuals from low socioeconomic groups. This approach, we believe, offers an exciting opportunity to optimize trial efficiency and create future designs to maximize the benefit of interventions in an equitable and sustainable way.

## Author Contributions

NS-D, AB, and KL wrote the manuscript. RdlT conceived, wrote and critically revised the manuscript. All authors read and approved the manuscript.

### Conflict of Interest Statement

The authors declare that the research was conducted in the absence of any commercial or financial relationships that could be construed as a potential conflict of interest.

## References

[1] Prince M Comas-Herrera A Knapp M Guerchet M Karagiannidou M World Alzheimer Report 2016 Improving Healthcare for People Living with Dementia. Coverage, Quality and Costs Now and in the Future. London (2016).

[2] Livingston G Sommerlad A Orgeta V Costafreda SG Huntley J Ames D. Dementia prevention, intervention, and care. Lancet. (2017) 390:2673–734. 10.1016/S0140-6736(17)31363-6 28735855

[3] World Health Organisation Dementia: Fact Sheet No. 362. WHO 2017. Available online at: http://www.who.int/mediacentre/factsheets/fs362/en/ (accessed March 26, 2018).

[4] Hankey G. Public health interventions for decreasing dementia risk. JAMA Neurol. (2018) 75:11–2. 10.1001/jamaneurol.2017.3303 29159408

[5] van de Rest O Berendsen AAM Haveman-nies A de Groot LC Rest O Van De Berendsen AAM. Dietary patterns, cognitive decline, and dementia : a systematic review. Adv Nutr Nutr. (2015) 6:154–68. 10.3945/an.114.007617 25770254 PMC4352174

[6] Miquel S Champ C Day J Aarts E Bahr BA Bakker M. Poor cognitive ageing: vulnerabilities, mechanisms and the impact of nutritional interventions. Ageing Res Rev. (2018) 42:40–55. 10.1016/j.arr.2017.12.004 29248758

[7] Loughrey D. Lavecchia S Brennan S Lawlor B Kelly M. The impact of the mediterranean diet on the cognitive functioning of healthy older adults: a systematic review and meta-analysis. Adv Nutr. (2017) 8:571–86. 10.3945/an.117.015495 28710144 PMC5502874

[8] Galvin JE. Prevention of alzheimer's disease: lessons learned and applied. J Am Geriatr Soc. (2017) 65:2128–33. 10.1111/jgs.14997 28766695 PMC5937943

[9] Ferry M Coley N Andrieu S Bonhomme C Caubere JP Cesari M. How to design nutritional intervention trials to populations and apply for efficacy claims : a statement from the international academy on nutrition and aging task force. J Nutr Heal Aging. (2013) 17:619–23. 10.1007/s12603-013-0350-y 23933873 PMC4312656

[10] Figueira I Menezes R Macedo D Costa I dos Santos CN. Polyphenols beyond barriers: a glimpse into the brain. Curr Neuropharmacol. (2017) 15:562–94. 10.2174/1570159X14666161026151545 27784225 PMC5543676

[11] Schelke MW Hackett K Chen JL Shih C Shum J Montgomery ME. Nutritional interventions for Alzheimer's prevention: a clinical precision medicine approach. Ann N Y Acad Sci. (2017) 1367:50–6. 10.1111/nyas.13070 27116241 PMC5674531

[12] de Roos B Brennan L. Personalised interventions—A precision approach for the next generation of dietary intervention studies. Nutrients. (2017) 9:1–9. 10.3390/nu9080847 28792454 PMC5579640

[13] Hampel H O'Bryant SE Durrleman S Younesi E Rojkova K Escott-Price V. A precision medicine initiative for alzheimer's disease: the road ahead to biomarker-guided integrative disease modeling. Climacteric. (2017) 20:107–18. 10.1080/13697137.2017.1287866 28286989

[14] Schulz KF Altman DG Moher D CONSORT Group. CONSORT 2010 statement: updated guidelines for reporting parallel group randomised trials. BMJ. (2010) 340:c332. 10.1136/bmj.c332 20332509 PMC2844940

[15] Lamont A Lyons MD Jaki T Stuart E Feaster DJ Tharmaratnam K. Identification of predicted individual treatment effects in randomized clinical trials. Stat Methods Med Res. (2016) 27:142–57. 10.1177/0962280215623981 26988928

[16] Kennedy-Martin T Curtis S Faries D Robinson S Johnston J. A literature review on the representativeness of randomized controlled trial samples and implications for the external validity of trial results. Trials. (2015) 16:495. 10.1186/s13063-015-1023-4 26530985 PMC4632358

[17] Shamseer L Sampson M Bukutu C Schmid CH Nikles J Tate R. CONSORT extension for reporting N-of-1 trials (CENT) 2015: explanation and elaboration. BMJ. (2015) 350:h1793. 10.1136/bmj.h1793 25976162

[18] Willke RJ Zheng Z Subedi P Althin R Mullins CD. From concepts, theory, and evidence of heterogeneity of treatment effects to methodological approaches: a primer. BMC Med Res Methodol. (2012) 12:1. 10.1186/1471-2288-12-185 23234603 PMC3549288

[19] Winblad B Amouyel P Andrieu S Ballard C Brayne C Brodaty H. Defeating Alzheimer's disease and other dementias: a priority for European science and society. Lancet Neurol. (2016) 15:455–532. 10.1016/S1474-4422(16)00062-4 26987701

[20] Duan N Eslick G Kaplan H Kravitz R Larson E Pace W Design and Implementation of N-of-1 Trials: A User's Guide. Agency of Health Care Research Quality (2014).

[21] OCEBM Levels of Evidence Working Group The Oxford 2011 Levels of Evidence. Oxford Centre for Evidence-Based Medicine. Available online at: http://www.cebm.net/index.aspx?o=5653

[22] Perdices M Tate RL. Single-subject designs as a tool for evidence-based clinical practice: are they unrecognised and undervalued? Neuropsychol Rehabil. (2009) 19:904–27. 10.1080/09602010903040691 19657974

[23] Edgington ES Statistics and single case analysis. Prog Behav Modif. (1984) 16:83–119. 10.1016/B978-0-12-535616-9.50008-0

[24] Weinreich SS Vrinten C Kuijpers MR Lipka AF Schimmel KJM Van Zwet EW. Aggregated N-of-1 trials for unlicensed medicines for small populations: an assessment of a trial with ephedrine for myasthenia gravis. Orphanet J Rare Dis. (2017) 12:88. 10.1186/s13023-017-0636-y 28494776 PMC5427624

[25] Chen X Chen P. A comparison of four methods for the analysis of n-of-1 trials. PLoS ONE. (2014) 9:e87752. 10.1371/journal.pone.0087752 24503561 PMC3913644

[26] Guyatt G Sackett D Adachi J Roberts R Chong J Rosenbloom D. A clinician's guide for conducting randomized trials in individual patients. CMAJ. (1988) 139:497–503. 3409138 PMC1268200

[27] Zucker DR Ruthazer R Schmid CH Feuer JM Fischer PA Kieval RI. Lessons learned combining N-of-1 trials to assess fibromyalgia therapies. J Rheumatol. (2006) 33:2069–77. 17014022

[28] Yelland MJ Nikles CJ McNairn N Del Mar CB Schluter PJ Brown RM. Celecoxib compared with sustained-release paracetamol for osteoarthritis: a series of n-of-1 trials. Rheumatology. (2007) 46:135–40. 10.1093/rheumatology/kel195 16777855

[29] Yelland MJ Poulos CJ Pillans PI Bashford GM Nikles CJ Sturtevant JM. N-of-1 randomized trials to assess the efficacy of gabapentin for chronic neuropathic pain. Pain Med. (2009) 10:754–61. 10.1111/j.1526-4637.2009.00615.x 19453961

[30] Nathan PC Tomlinson G Dupuis LL Greenberg ML Ota S Bartels U. A pilot study of ondansetron plus metopimazine vs. ondansetron monotherapy in children receiving highly emetogenic chemotherapy: a bayesian randomized serial N-of-1 trials design. Support Care Cancer. (2006) 14:268–76. 10.1007/s00520-005-0875-7 16052316

[31] Vieira R McDonald S Araújo-Soares V Sniehotta FF Henderson R. Dynamic modelling of n-of-1 data: powerful and flexible data analytics applied to individualised studies. Health Psychol Rev. (2017) 11:222–34. 10.1080/17437199.2017.1343680 28629262

[32] Cornu C Kassai B Fisch R Chiron C Alberti C Guerrini R. Experimental designs for small randomised clinical trials: an algorithm for choice. Orphanet J Rare Dis. (2013) 8:48. 10.1186/1750-1172-8-48 23531234 PMC3635911

[33] Ordovas JM Ferguson LR Tai ES Mathers JC. Personalised nutrition and health. Bmj 2018:bmj.k2173. 10.1136/bmj.k2173 29898881 PMC6081996

[34] Maher M Pooler AM Kaput J Kussmann M A systems approach to personalised nutrition: report on the keystone symposium “human nutrition, environment and health.” Appl Transl Genomics. (2016) 10:16–8. 10.1016/j.atg.2016.08.001 PMC5025480 27668171

[35] Nielsen J. Systems biology of metabolism: a driver for developing personalized and precision medicine. Cell Metab. (2017) 25:572–9. 10.1016/j.cmet.2017.02.002 28273479

[36] Hood L Friend SH. Predictive, personalized, preventive, participatory (P4) cancer medicine. Nat Rev Clin Oncol. (2011) 8:184–7. 10.1038/nrclinonc.2010.227 21364692

[37] Vitali F Li Q Schissler AG Berghout J Kenost C Lussier YA. Developing a ‘personalome’ for precision medicine: emerging methods that compute interpretable effect sizes from single-subject transcriptomes. Brief Bioinform. (2017) 20:789–805. 10.1093/bib/bbx149 29272327 PMC6585155

[38] Collins F Varmus H. A new initiative on precision medicine. N Engl J Med. (2015) 372:793–5. 10.1056/NEJMp1500523 25635347 PMC5101938

[39] Jack CR Knopman DS Jagust WJ Shaw LM Aisen PS Weiner MW. Hypothetical model of dynamic biomarkers of the Alzheimer's pathological cascade. Lancet Neurol. (2010) 9:119–28. 10.1016/S1474-4422(09)70299-6 20083042 PMC2819840

[40] Dubois B Hampel H Feldman HH Scheltens P Aisen P Andrieu S. Preclinical Alzheimer's disease: definition, natural history, and diagnostic criteria. Alzheimers Dement. (2016) 12:292–323. 10.1016/j.jalz.2016.02.002 27012484 PMC6417794

[41] Yanker B Lu T Loerch P The aging brain. Annu Rev Pathol Mech Dis. (2008) 3:41–66. 10.1146/annurev.pathmechdis.2.010506.092044 18039130

[42] Ávila-Villanueva M Fernández-Blázquez MA. Subjective cognitive decline as a preclinical marker for alzheimer's disease: the challenge of stability over time. Front Aging Neurosci. (2017) 9:1–3. 10.3389/fnagi.2017.00377 29201004 PMC5696596

[43] Carter MD Simms GA Weaver DF. The development of new therapeutics for Alzheimer's disease. Clin Pharmacol Ther. (2010) 88:475–86. 10.1038/clpt.2010.165 20811351

[44] Jessen F Amariglio RE Van Boxtel M Breteler M Ceccaldi M Chételat G A conceptual framework for research on subjective cognitive decline in preclinical Alzheimer's disease. Alzheimers Dement. (2014) 10:844–52. 10.1016/j.jalz.2014.01.001 24798886 PMC4317324

[45] Zhang T Liu S Zhang Y Guan Y Wang X Zhao L. Apolipoprotein e e4 allele is associated with subjective cognitive decline: a meta-analysis. Neuroepidemiology. (2017) 49:165–73. 10.1159/000482018 29169179

[46] Montejo Carrasco P Montenegro-Peña M López-Higes R Estrada E Prada Crespo D Montejo Rubio C. Subjective memory complaints in healthy older adults: fewer complaints associated with depression and perceived health, more complaints also associated with lower memory performance. Arch Gerontol Geriatr. (2017) 70:28–37. 10.1016/j.archger.2016.12.007 28039781

[47] Cheng Y Chen T Chiu M. From mild cognitive impairment to subjective cognitive decline: conceptual and methodological evolution. Neuropsychiatr Dis Treat. (2017) 13:491–8. 10.2147/NDT.S123428 28243102 PMC5317337

[48] Petersen RC Caracciolo B Brayne C Gauthier S Jelic V Fratiglioni L. Mild cognitive impairment: a concept in evolution. J Intern Med. (2014) 275:214–28. 10.1111/joim.12190 24605806 PMC3967548

[49] Dangour AD Allen E Richards M Whitehouse P Uauy R Design considerations in long-term intervention studies for the prevention of cognitive decline or dementia. Nutr Rev. (2010) 68:S16–21. 10.1111/j.1753-4887.2010.00330.x 20946363

[50] Whitehouse PJ. Mild cognitive impairment—a confused concept? Nat Clin Pract Neurol. (2007) 3:62–3. 10.1038/ncpneuro0403 17279077

[51] Dubois B Epelbaum S Nyasse F Bakardjian H Gagliardi G Uspenskaya O. Cognitive and neuroimaging features and brain β-amyloidosis in individuals at risk of Alzheimer's disease (INSIGHT-preAD): a longitudinal observational study. Lancet Neurol. (2018) 17:335–46. 10.1016/S1474-4422(18)30029-2 29500152

[52] Balasubramanian AB Kawas CH Peltz CB Brookmeyer R Corrada MM. Alzheimer disease pathology and longitudinal cognitive performance in the oldest-old with no dementia. Neurology. (2012) 79:915–21. 10.1212/WNL.0b013e318266fc77 22895581 PMC3425842

[53] Corrada MM Berlau DJ Kawas CH. A population-based clinicopathological study in the oldest-old: the 90+ study. Curr Alzheimer Res. (2012) 9:709–17. 10.2174/156720512801322537 22471863 PMC3409303

[54] Ferrari C Xu WL Wang HX Winblad B Sorbi S Qiu C. How can elderly apolipoprotein E ε4 carriers remain free from dementia? Neurobiol Aging. (2013) 34:13–21. 10.1016/j.neurobiolaging.2012.03.003 22503000

[55] Raz N Lindenberger U Rodrigue KM Kennedy KM Head D Williamson A. Regional brain changes in aging healthy adults: general trends, individual differences and modifiers. Cereb Cortex. (2005) 15:1676–89. 10.1093/cercor/bhi044 15703252

[56] Ghisletta P Rabbitt P Lunn M Lindenberger U Two thirds of the age-based changes in fluid and crystallized intelligence, perceptual speed, and memory in adulthood are shared. Intelligence. (2012) 40:260–8. 10.1016/j.intell.2012.02.008

[57] Cabeza R Albert M Belleville S Craik FIM Duarte A Grady CL Maintenance, reserve and compensation: the cognitive neuroscience of healthy ageing. Nat Rev Neurosci. (2018) 19:701–10. 10.1038/s41583-018-0068-2 30305711 PMC6472256

[58] Piras F Cherubini A Caltagirone C Spalletta G. Education mediates microstructural changes in bilateral hippocampus. Hum Brain Mapp (2011) 32:282–9. 10.1002/hbm.21018 20336658 PMC6870355

[59] Zhang J Zhou W Cassidy RM Su H Su Y Zhang X. Risk factors for amyloid positivity in older people reporting significant memory concern. Compr Psychiatry. (2018) 80:126–31. 10.1016/j.comppsych.2017.09.015 29091778

[60] Barulli D Stern Y. Efficiency, capacity, compensation, maintenance, plasticity: emerging concepts in cognitive reserve. Trends Cogn Sci. (2013) 17:502–9. 10.1016/j.tics.2013.08.012 24018144 PMC3840716

[61] Bialystok E Craik FIM Luk G. Bilingualism: consequences for mind and brain. Trends Cogn Sci. (2012) 16:240–50. 10.1016/j.tics.2012.03.001 22464592 PMC3322418

[62] Barha CK Liu-Ambrose T. Exercise and the aging brain: considerations for sex differences. Brain Plast. (2018) 4:53–63. 10.3233/BPL-180067 30564546 PMC6296261

[63] Prakash RS Voss MW Erickson KI Kramer AF. Physical activity and cognitive vitality. Annu Rev Psychol. (2015) 66:769–97. 10.1146/annurev-psych-010814-015249 25251492

[64] Vaci N Gula B Bilalić M. Is age really cruel to experts? Compensatory effects of activity. Psychol Aging. (2015) 30:740–54. 10.1037/pag0000056 26523694

[65] Ten Brinke LF Bolandzadeh N Nagamatsu LS Liang Hsu C Davis JC Miran-Khan K Aerobic exercise increases hippocampal volume in older women with probable mild cognitive impairment: a 6-month randomized controlled trial. Br J Sports Med. (2015) 49:248–54. 10.1136/bjsports-2013-093184 24711660 PMC4508129

[66] Aartsen MJ Cheval B Sieber S Van der Linden BW Gabriel R Courvoisier DS. Advantaged socioeconomic conditions in childhood are associated with higher cognitive functioning but stronger cognitive decline in older age. Proc Natl Acad Sci USA. (2019) 116:5478–86. 10.1073/pnas.1807679116 30804194 PMC6431198

[67] Arenaza-Urquijo EM Bejanin A Gonneaud J Wirth M La Joie R Mutlu J. Association between educational attainment and amyloid deposition across the spectrum from normal cognition to dementia: neuroimaging evidence for protection and compensation. Neurobiol Aging. (2017) 59:72–9. 10.1016/j.neurobiolaging.2017.06.016 28764930

[68] Driscoll I Troncoso J Asymptomatic Alzheimer's disease: a prodrome or a state of resilience? Curr Alzheimer Res. (2011) 8:330–5. 21222594 10.2174/156720511795745348 PMC3286868

[69] Brickman AM Siedlecki KL Muraskin J Manly JJ Luchsinger JA Yeung L-K. White matter hyperintensities and cognition: testing the reserve hypothes. Neurobiol Aging. (2011) 32:1588–98. 10.1016/j.neurobiolaging.2009.10.013 19926168 PMC2891625

[70] Landau SM Marks SM Mormino EC Rabinovici GD Oh H O JP. Association of lifetime cognitive engagement and low β-amyloid deposition. Arch Neurol. (2012) 69:623–9. 10.1001/archneurol.2011.2748 22271235 PMC3747737

[71] Soldan A Pettigrew C Li S Wang M-C Moghekar A Selnes OA Relationship of cognitive reserve and CSF biomarkers to emergence of clinical symptoms in preclinical Alzheimer's Disease. Neurobiol Aging. (2013) 34:2827–34. 10.1016/j.neurobiolaging.2013.06.017 23916061 PMC3823238

[72] Schikowski T Vossoughi M Vierkötter A Schulte T Teichert T Sugiri D. Association of air pollution with cognitive functions and its modification by APOE gene variants in elderly women. Environ Res. (2015) 142:10–6. 10.1016/j.envres.2015.06.009 26092807

[73] Wilker EH Preis SR Beiser AS Wolf PA Au R Kloog I. Long-term exposure to fine particulate matter, residential proximity to major roads and measures of brain structure. Stroke. (2015) 46:1161–6. 10.1161/STROKEAHA.114.008348 25908455 PMC4414870

[74] Miller JW Harvey DJ Beckett LA Green R Farias ST Reed BR. Vitamin D status and rates of cognitive decline in a multiethnic cohort of older adults. JAMA Neurol. (2016) 72:1295–303. 10.1001/jamaneurol.2015.2115 26366714 PMC5023277

[75] Kishimoto H Ohara T Hata J Ninomiya T Yoshida D Mukai N. The long-term association between physical activity and risk of dementia in the community: the Hisayama Study. Eur J Epidemiol. (2016) 31:267–74. 10.1007/s10654-016-0125-y 26857126

[76] De Bruijn RFAG Schrijvers EMC De Groot KA Witteman JCM Hofman A Franco OH. The association between physical activity and dementia in an elderly population: the rotterdam study. Eur J Epidemiol. (2013) 28:277–83. 10.1007/s10654-013-9773-3 23385659

[77] Hardman RJ Kennedy G Macpherson H Scholey AB Pipingas A. Adherence to a mediterranean-style diet and effects on cognition in adults: a qualitative evaluation and systematic review of longitudinal and prospective trials. Front Nutr. (2016) 3:1–13. 10.3389/fnut.2016.00022 27500135 PMC4956662

[78] Fenech M. Vitamins Associated with brain aging, mild cognitive impairment, and alzheimer disease: biomarkers, epidemiological and experimental evidence, plausible mechanisms, and knowledge gaps. Adv Nutr An Int Rev J. (2017) 8:958–70. 10.3945/an.117.015610 29141977 PMC5682999

[79] Abate G Marziano M Rungratanawanich W Memo M Uberti D. Nutrition and AGE-ing: focusing on Alzheimer's Disease. Oxid Med Cell Longev. (2017) 2017:7039816. 10.1155/2017/7039816 28168012 PMC5266861

[80] Rodríguez-Morató J Xicota L Fitó M Farré M Dierssen M De La Torre R. Potential role of olive oil phenolic compounds in the prevention of neurodegenerative diseases. Molecules. (2015) 20:4655–80. 10.3390/molecules20034655 25781069 PMC6272603

[81] Caruana M Cauchi R Vassallo N. Putative role of red wine polyphenols against brain pathology in alzheimer's and parkinson's disease. Front Nutr. (2016) 3:31. 10.3389/fnut.2016.00031 27570766 PMC4981604

[82] Martínez-Huélamo M Rodríguez-Morató J Boronat A de la Torre R. Modulation of Nrf2 by olive oil and wine polyphenols and neuroprotection. Antioxidants. (2017) 6:73. 10.3390/antiox6040073 28954417 PMC5745483

[83] Pasinetti GM Novel role of red wine-derived polyphenols in the prevention of Alzheimer's disease dementia and brain pathology: experimental approaches and clinical implications. Planta Med. (2012) 78:1614–20. 10.1055/s-0032-1315377 23023952

[84] Murphy T Dias GP Thuret S. Effects of diet on brain plasticity in animal and human studies: mind the gap. Neural Plast. (2014) 2014:1–32. 10.1155/2014/563160 24900924 PMC4037119

[85] Kean RJ Lamport DJ Dodd GF Freeman JE Williams CM Ellis JA. Chronic consumption of flavanone-rich orange juice is associated with cognitive benefits : an 8-wk, randomized, double-blind, placebo-controlled trial in healthy older adults. Am J Clin Nutr. (2015) 101:506–14. 10.3945/ajcn.114.088518 25733635

[86] Brickman A Khan U Provenzano F Yeung L Suzuki W Schroeter H. Enhancing dentate gyrus function with dietary flavanols improves cognition in older adults. Nat Neurosci. (2014) 17:1798–803. 10.1038/nn.3850 25344629 PMC4940121

[87] Mastroiacovo D Kwik-uribe C Grassi D Necozione S Raffaele A Pistacchio L. Cocoa flavanol consumption improves cognitive function, blood pressure control, and metabolic profile in elderly subjects: the Cocoa, Cognition, and Aging (CoCoA) Study - a randomized controlled trial. Am J Clin Nutr. (2015) 101:538–48. 10.3945/ajcn.114.092189 25733639 PMC4340060

[88] Krikorian R Shidler MD Nash TA Kalt W Vinqvist-tymchuk MR Shukitt-hale B. Blueberry supplementation improves memory in older adults. J Agric Food Chem. (2010) 58:3996–4000. 10.1021/jf9029332 20047325 PMC2850944

[89] Miller MG Hamilton DA Joseph JA Shukitt-Hale B. Dietary blueberry improves cognition among older adults in a randomized, double-blind, placebo-controlled trial. Eur J Nutr. (2018) 57:1169–80. 10.1007/s00394-017-1400-8 28283823

[90] Bowtell JL Aboo-Bakkar Z Conway ME Adlam A-LR Fulford J. Enhanced task-related brain activation and resting perfusion in healthy older adults after chronic blueberry supplementation. Appl Physiol Nutr Metab. (2017) 42:773–9. 10.1139/apnm-2016-0550 28249119

[91] Witte A V Kerti L Margulies DS Floel A. Effects of resveratrol on memory performance, hippocampal functional connectivity, and glucose metabolism in healthy older adults. J Neurosci. (2014) 34:7862–70. 10.1523/JNEUROSCI.0385-14.2014 24899709 PMC6608268

[92] Krikorian R Boespflug EL Fleck DE Stein AL Wightman JD Shidler MD. Concord grape juice supplementation and neurocognitive function in human aging. J Agric Food Chem. (2012) 60:5736–42. 10.1021/jf300277g 22468945

[93] Boespflug EL Eliassen JC Dudley JA Shidler MD Kalt W Summer SS. Enhanced neural activation with blueberry supplementation in mild cognitive impairment. Nutr Neurosci. (2018) 21:297–305. 10.1080/1028415X.2017.1287833 28221821 PMC6093614

[94] Park S-K Jung I-C Lee WK Lee YS Park HK A combination of green tea extract and l-theanine improves memory and attention in subjects with mild cognitive impairment: a double-blind placebo-controlled Study Sang-Ki. J Med Food. (2011) 14:334–43. 10.1089/jmf.2009.1374 21303262

[95] Kent K Charlton K Roodenrys S Batterham M Potter J Traynor V. Consumption of anthocyanin-rich cherry juice for 12 weeks improves memory and cognition in older adults with mild-to-moderate dementia. Eur J Nutr. (2017) 56:333–41. 10.1007/s00394-015-1083-y 26482148

[96] Lee J Torosyan N Silverman DH. Examining the impact of grape consumption on brain metabolism and cognitive function in patients with mild decline in cognition: a double-blinded placebo controlled pilot study. Exp Gerontol. (2017) 87:121–8. 10.1016/j.exger.2016.10.004 27856335

[97] Ide K Yamada H Takuma N Park M Wakamiya N Nakase J. Green tea consumption affects cognitive dysfunction in the elderly: a pilot study. Nutrients. (2014) 6:4032–42. 10.3390/nu6104032 25268837 PMC4210905

[98] Dodge H Zitzelberger T Oken B Howieson D Kaye J. A randomized placebo-controlled trial of Ginkgo biloba for the prevention of cognitive decline. Neurology. (2009) 70:1809–17. 10.1212/01.wnl.0000303814.13509.db 18305231 PMC2639649

[99] Dekosky ST Williamson JD Fitzpatrick AL Kronmal RA Ives DG Saxton JA. Ginkgo biloba for prevention of dementia: a randomized controlled trial. J Am Med Assoc. (2008) 300:2253–62. 10.1001/jama.2008.683 19017911 PMC2823569

[100] Mathis CA Kuller LH Klunk WE Snitz BE Price JC Weissfeld LA. *In vivo* assessment of amyloid-β deposition in nondemented very elderly subjects. Ann Neurol. (2013) 73:751–61. 10.1002/ana.23797 23596051 PMC3725727

[101] Ringman JM Frautschy SA Teng E Begum AN Bardens J Beigi M. Oral curcumin for Alzheimer's disease: tolerability and efficacy in a 24-week randomized, double blind, placebo-controlled study. Alzheimer's Res Ther. (2012) 4:43. 10.1186/alzrt146 23107780 PMC3580400

[102] Vellas B Coley N Ousset PJ Berrut G Dartigues JF Dubois B. Long-term use of standardised ginkgo biloba extract for the prevention of Alzheimer's disease (GuidAge): a randomised placebo-controlled trial. Lancet Neurol. (2012) 11:851–9. 10.1016/S1474-4422(12)70206-5 22959217

[103] Turner RS Thomas RG Craft S Van Dyck CH Mintzer J Reynolds BA. A randomized, double-blind, placebo-controlled trial of resveratrol for Alzheimer disease. Neurology. (2015) 85:1383–91. 10.1212/WNL.0000000000002035 26362286 PMC4626244

[104] Molino S Dossena M Buonocore D Ferrari F Venturini L Ricevuti G. Polyphenols in dementia: from molecular basis to clinical trials. Life Sci. (2016) 161:69–77. 10.1016/j.lfs.2016.07.021 27493077

[105] Huhn S Masouleh SK Villringer A Witte AV. Components of a Mediterranean diet and their impact on cognitive functions in aging. Front Aging Neurosci. (2015) 7:1–10. 10.3389/fnagi.2015.00132 26217224 PMC4495334

[106] Espín JC González-Sarrías A Tomás-Barberán FA. The gut microbiota: a key factor in the therapeutic effects of (poly)phenols. Biochem Pharmacol. (2017) 139:82–93. 10.1016/j.bcp.2017.04.033 28483461

[107] Kang C Zhang Y Zhu X Liu K Wang X Chen M. Healthy subjects differentially respond to dietary capsaicin correlating with specific gut enterotypes. J Clin Endocrinol Metab. (2016) 101:4681–9. 10.1210/jc.2016-2786 27676396

[108] Lagkouvardos I Kläring K Heinzmann SS Platz S Scholz B Engel K-H. Gut metabolites and bacterial community networks during a pilot intervention study with flaxseeds in healthy adult men. Mol Nutr Food Res. (2015) 59:1614–28. 10.1002/mnfr.201500125 25988339

[109] Gomez-Pinilla F Tyagi E. Diet and cognition: interplay between cell metabolism and neuronal plasticity. Curr Opin Clin Nutr Metab Care. (2013) 16:726–33. 10.1097/MCO.0b013e328365aae3 24071781 PMC4005410

[110] Hooijmans CR Rutters F Dederen PJ Gambarota G Veltien A van Groen T. Changes in cerebral blood volume and amyloid pathology in aged Alzheimer APP/PS1 mice on a docosahexaenoic acid (DHA) diet or cholesterol enriched Typical Western Diet (TWD). Neurobiol Dis. (2007) 28:16–29. 10.1016/j.nbd.2007.06.007 17720508

[111] Oksman M Iivonen H Hogyes E Amtul Z Penke B Leenders I. Impact of different saturated fatty acid, polyunsaturated fatty acid and cholesterol containing diets on beta-amyloid accumulation in APP/PS1 transgenic mice. Neurobiol Dis. (2006) 23:563–72. 10.1016/j.nbd.2006.04.013 16765602

[112] EFSA NDA (EFSA Panel on Dietetic Products Nutrition and Allergies 2016) Scientific opinion and DHA and improvement of memory function: evaluation of a health claim pursuant to Article 13(5) of Regulation (EC) No 1924/2006. EFSA J. (2016) 14:4455 10.2903/j.efsa.2016.4455 PMC13151543 42109460

[113] Nilsson A Radeborg K Salo I Björck I. Effects of supplementation with n-3 polyunsaturated fatty acids on cognitive performance and cardiometabolic risk markers in healthy 51 to 72 years old subjects: a randomized controlled cross-over study. Nutr J. (2012) 11:1–9. 10.1186/1475-2891-11-99 23173831 PMC3564898

[114] Dangour AD Allen E Elbourne D Fasey N Fletcher AE Hardy P Effect of 2-y n23 long-chain polyunsaturated fatty acid supplementation on cognitive function in older people: a randomized, double-blind, controlled trial. Am J Clin Nutr. (2010) 91:1725–32. 10.3945/ajcn.2009.29121 20410089

[115] Van de Rest O Geleijnse JM Kok FJ van Staveren WA Dullemeijer C. Effect of fish oil on cognitive performance in older subjects. Neurology. (2008) 71:430–8. 10.1212/01.wnl.0000324268.45138.86 18678826

[116] Witte AV Kerti L Hermannstädter HM Fiebach JB Schreiber SJ Schuchardt JP. Long-chain omega-3 fatty acids improve brain function and structure in older adults. Cereb Cortex. (2014) 24:3059–68. 10.1093/cercor/bht163 23796946

[117] Yurko-Mauro K McCarthy D Rom D Nelson EB Ryan AS Blackwell A. Beneficial effects of docosahexaenoic acid on cognition in age-related cognitive decline. Alzheimer's Dement. (2010) 6:456–64. 10.1016/j.jalz.2010.01.013 20434961

[118] Tokuda H Sueyasu T Kontani M Kawashima H Shibata H Koga Y. Low doses of long-chain polyunsaturated fatty acids affect cognitive function in elderly japanese men: a randomized controlled trial. J Oleo Sci J Oleo Sci. (2015) 64:633–44. 10.5650/jos.ess15009 25891115

[119] Chiu CC Su KP Cheng TC Liu HC Chang CJ Dewey ME. The effects of omega-3 fatty acids monotherapy in Alzheimer's disease and mild cognitive impairment: a preliminary randomized double-blind placebo-controlled study. Prog Neuro-Psychopharmacology Biol Psychiatry. (2008) 32:1538–44. 10.1016/j.pnpbp.2008.05.015 18573585

[120] Freund-Levi Y Eriksdotter-Jonhagen M Cederholm T Basun H Faxen-Irving G Garlind A. ω-3 Fatty acid treatment in 174 patients with mild to moderate alzheimer disease: OmegAD study. Arch Neurol. (2006) 63:1402–8. 10.1001/archneur.63.10.1402 17030655

[121] Quinn JF Raman R Thomas RG Yurko-Mauro K Nelson EB Van Dyck C. Docosahexaenoic acid supplementation and cognitive decline in Alzheimer disease: a randomized trial. JAMA. (2010) 304:1903–11. 10.1001/jama.2010.1510 21045096 PMC3259852

[122] Bo Y Zhang X Wang Y You J Cui H Zhu Y. The n-3 polyunsaturated fatty acids supplementation improved the cognitive function in the Chinese elderly with mild cognitive impairment: a double-blind randomized controlled trial. Nutrients. (2017) 9:1–11. 10.3390/nu9010054 28075381 PMC5295098

[123] Lee LK Shahar S Chin A-V Yusoff NAM. Docosahexaenoic acid-concentrated fish oil supplementation in subjects with mild cognitive impairment (MCI): a 12-month randomised, double-blind, placebo-controlled trial. Psychopharmacology. (2013) 225:605–12. 10.1007/s00213-012-2848-0 22932777

[124] Mazereeuw G Lanctôt KL Chau SA Swardfager W Herrmann N. Effects of omega-3 fatty acids on cognitive performance: a meta-analysis. Neurobiol Aging. (2012) 33:1482.e17-1482.e29. 10.1016/j.neurobiolaging.2011.12.014 22305186

[125] Burckhardt M Herke M Wustmann T Watzke S Langer G Fink A. Omega-3 fatty acids for the treatment of dementia. Cochrane Database Syst Rev. (2016) 4:CD009002. 10.1002/14651858.CD009002.pub3 27063583 PMC7117565

[126] Chilton F Dutta R Reynolds L Sergeant S Mathias R Seeds M. Precision nutrition and Omega-3 polyunsaturated fatty acids: a case for personalized supplementation approaches for the prevention and management of human diseases. Nutrients. (2017) 9:1165. 10.3390/nu9111165 29068398 PMC5707637

[127] Moore K Hughes CF Ward M Hoey L McNulty H. Diet, nutrition and the ageing brain: current evidence and new directions. Proc Nutr Soc. (2018) 77:152–63. 10.1017/S0029665117004177 29316987

[128] Jayedi A Rashidy-Pour A Shab-Bidar S Vitamin D status and risk of dementia and Alzheimer's disease: a meta-analysis of dose-response. Nutr Neurosci. (2018) 15:1–10. 10.1080/1028415X.2018.1436639 29447107

[129] Holick MF Binkley NC Bischoff-Ferrari HA Gordon CM Hanley DA Heaney RP. Evaluation, treatment, and prevention of vitamin D deficiency: an endocrine society clinical practice guideline. J Clin Endocrinol Metab. (2011) 96:1911–30. 10.1210/jc.2011-0385 21646368

[130] Pettersen JA. Does high dose vitamin D supplementation enhance cognition?: a randomized trial in healthy adults. Exp Gerontol. (2017) 90:90–7. 10.1016/j.exger.2017.01.019 28167237

[131] Annweiler C Llewellyn DJ Beauchet O. Low serum vitamin D concentrations in Alzheimer's disease: a systematic review and meta-analysis. J Alzheimer's Dis. (2013) 33:659–74. 10.3233/JAD-2012-121432 23042216

[132] Rossom R Espeland M Manson J Dysken M Johnson K Lane D. Calcium and vitamin D supplementation and cognitive impairment in the women's health initiative. J Am Geriatr Soc. (2012) 60:2197–205. 10.1111/jgs.12032 23176129 PMC3521077

[133] Aspell N Lawlor B O'Sullivan M Is there a role for vitamin D in supporting cognitive function as we age? Proc Nutr Soc. (2017) 25:124–34. 10.1017/S0029665117004153 29233204

[134] Schelke MW Attia P Palenchar DJ Kaplan B Mureb M Ganzer CA. Mechanisms of risk reduction in the clinical practice of Alzheimer's disease prevention. Front Aging Neurosci. (2018) 10:1–14. 10.3389/fnagi.2018.00096 29706884 PMC5907312

[135] Smith AD Refsum H. Homocysteine, B vitamins, and cognitive impairment. Annu Rev Nutr. (2016) 36:211–39. 10.1146/annurev-nutr-071715-050947 27431367

[136] Durga J van Boxtel MP Schouten EG Kok FJ Jolles J Katan MB. Effect of 3-year folic acid supplementation on cognitive function in older adults in the FACIT trial: a randomised, double blind, controlled trial. Lancet. (2007) 369:208–16. 10.1016/S0140-6736(07)60109-3 17240287

[137] Malouf R Evans JG Reem M John GE. Folic acid with or without vitamin B12 for the prevention and treatment of healthy elderly and demented people. Cochrane Database Syst Rev. (2008). 4:CD004514. 10.1002/14651858.CD004514.pub2 18843658 PMC12926861

[138] Travica N Ried K Sali A Scholey A Hudson I Pipingas A. Vitamin c status and cognitive function: a systematic review. Nutrients. (2017) 9:1–21. 10.3390/nu9090960 28867798 PMC5622720

[139] Monacelli F Acquarone E Giannotti C Borghi R Nencioni A. Vitamin C, aging and Alzheimer's disease. Nutrients. (2017) 9:670. 10.3390/nu9070670 28654021 PMC5537785

[140] Fillenbaum GG Kuchibhatla MN Hanlon JT Artz MB Pieper CF Schmader KE Dementia and Alzheimer's disease in community-dwelling elders taking vitamin C and/or vitamin E. Ann Pharmacother. (2005) 39:2009–14. 10.1345/aph.1G280 16227448

[141] Boccardi V Baroni M Mangialasche F Mecocci P. Vitamin E family: role in the pathogenesis and treatment of Alzheimer's disease. Alzheimer's Dement Transl Res Clin Interv. (2016) 2:182–91. 10.1016/j.trci.2016.08.002 29067305 PMC5651353

[142] Dysken MW Sano M Asthana S Vertrees JE Pallaki M Llorente M A randomized, clinical trial of vitamin E and memantine in alzheimer's disease (TEAM-AD). JAMA. (2014) 311:33–44. 10.1001/jama.2013.282834 24381967 PMC4109898

[143] Farina N Llewellyn D Isaac MGEKN Tabet N. Vitamin E for Alzheimer's dementia and mild cognitive impairment. Cochrane Database Syst Rev. (2017) 4:CD002854. 10.1002/14651858.CD002854.pub5 28418065 PMC6478142

[144] Takasaki J Ono K Yoshiike Y Hirohata M Ikeda T Morinaga A. Vitamin A has anti-oligomerization effects on amyloid-β *in vitro*. J Alzheimer's Dis. (2014) 27:271–80. 10.3233/JAD-2011-110455 21811022

[145] Cansev M van Wijk N Turkyilmaz M Orhan F Sijben JWC Broersen LM. A specific multi-nutrient enriched diet enhances hippocampal cholinergic transmission in aged rats. Neurobiol Aging. (2015) 36:344–51. 10.1016/j.neurobiolaging.2014.07.021 25146455

[146] Wiesmann M Zerbi V Jansen D Haast R Lütjohann D Broersen LM. A dietary treatment improves cerebral blood flow and brain connectivity in aging apoE4 mice. Neural Plast. (2016) 2016:9–11. 10.1155/2016/6846721 27034849 PMC4806294

[147] Soininen H Solomon A Visser PJ Hendrix SB Blennow K Kivipelto M. 24-month intervention with a specific multinutrient in people with prodromal Alzheimer's disease (LipiDiDiet): a randomised, double-blind, controlled trial. Lancet Neurol. (2017) 16:965–75. 10.1016/S1474-4422(17)30332-0 29097166 PMC5697936

[148] Small BJ Rawson KS Martin C Eisel SL Sanberg CD McEvoy CL. Nutraceutical Intervention Improves Older Adults' Cognitive Functioning. Rejuvenation Res. (2014) 17:27–32. 10.1089/rej.2013.1477 24134194 PMC4047846

[149] Baleztena J Ruiz-Canela M Sayon-Orea C Pardo M Añorbe T Gost JI. Association between cognitive function and supplementation with omega-3 PUFAs and other nutrients in 75 years old patients: a randomized multicenter study. PLoS ONE. (2018) 13:1–15. 10.1371/journal.pone.0193568 29579102 PMC5868762

[150] McNamara RK Kalt W Shidler MD McDonald J Summer SS Stein AL. Cognitive response to fish oil, blueberry, and combined supplementation in older adults with subjective cognitive impairment. Neurobiol Aging. (2018) 64:147–56. 10.1016/j.neurobiolaging.2017.12.003 29458842 PMC5822748

[151] Bjelakovic G Nikolova D Ll G Rg S Gluud C Bjelakovic G Antioxidant supplements for prevention of mortality in healthy participants and patients with various diseases. Cochrane Database Syst Rev. (2012) 3:CD007176 10.1002/14651858.CD007176.pub2 PMC8407395 22419320

[152] Milte CM McNaughton SA. Dietary patterns and successful ageing: a systematic review. Eur J Nutr. (2016) 55:423–50. 10.1007/s00394-015-1123-7 26695408 PMC4767865

[153] Vos T Alemu Abajobir A Hassen Abate K Abbafati C Abbas KM Abd-Allah F Global, regional, and national incidence, prevalence, and years lived with disability for 328 diseases and injuries for 195 countries, 1990-2016: a systematic analysis for the Global Burden of Disease Study 2016. Lancet. (2017) 390:1211–59. 10.1016/S0140-6736(17)32154-2 28919117 PMC5605509

[154] Trichopoulou A Martínez-González MA Tong TYN Forouhi NG Khandelwal S Prabhakaran D. Definitions and potential health benefits of the Mediterranean diet: views from experts around the world. BMC Med. (2014) 12:1–16. 10.1186/1741-7015-12-112 25055810 PMC4222885

[155] Frisardi V Panza F Seripa D Imbimbo BP Vendemiale G Pilotto A. Nutraceutical properties of mediterranean diet and cognitive decline: possible underlying mechanisms. J Alzheimer's Dis. (2010) 22:715–40. 10.3233/JAD-2010-100942 20858954

[156] Dinu M Pagliai G Casini A Sofi F. Mediterranean diet and multiple health outcomes: an umbrella review of meta-analyses of observational studies and randomised trials. Eur J Clin Nutr. (2018) 72:30–43. 10.1038/ejcn.2017.58 28488692

[157] Aridi Y Walker J Wright O. The association between the mediterranean dietary pattern and cognitive health: a systematic review. Nutrients. (2017) 9:674. 10.3390/nu9070674 28657600 PMC5537789

[158] Wu L Sun D. Adherence to Mediterranean diet and risk of developing cognitive disorders: an updated systematic review and meta-analysis of prospective cohort studies. Sci Rep. (2017) 7:41317. 10.1038/srep41317 28112268 PMC5256032

[159] Estruch R Ros E Salas-Salvadó J Covas M-I Corella D Arós F. Primary prevention of cardiovascular disease with a mediterranean diet. N Engl J Med. (2013) 368:1279–90. 10.1056/NEJMoa1200303 29897867

[160] Valls-Pedret C Sala-Vila A Serra-Mir M Corella D De La Torre R Martínez-González MÁ. Mediterranean diet and age-related cognitive decline: a randomized clinical trial. JAMA Intern Med. (2015) 175:1094–103. 10.1001/jamainternmed.2015.1668 25961184

[161] Martínez-Lapiscina EH Clavero P Toledo E Estruch R Salas-Salvadó J San Julián B. Mediterranean diet improves cognition: the PREDIMED-NAVARRA randomised trial. J Neurol Neurosurg Psychiatry. (2013) 84:1318–25. 10.1136/jnnp-2012-304792 23670794

[162] Petersson SD Philippou E. Mediterranean Diet, Cognitive function, and dementia: a systematic review of the evidence. Adv Nutr. (2016) 7:889–904. 10.3945/an.116.012138 27633105 PMC5015034

[163] Berendsen AAM van de Rest O Feskens EJM de Groot LCPGM Kang JH Grodstein F. The dietary approaches to stop hypertension diet, cognitive function, and cognitive decline in american older women. J Am Med Dir Assoc. (2017) 18:427–32. 10.1016/j.jamda.2016.11.026 28108204

[164] McEvoy CT Guyer H Langa KM Yaffe K. Neuroprotective diets are associated with better cognitive function: the health and retirement study. J Am Geriatr Soc. (2017) 65:1857–62. 10.1111/jgs.14922 28440854 PMC5633651

[165] Morris M Tangney C Wang Y Sacks F Barnes L Bennett D. MIND diet slows cognitive decline with aging. Alzheimers Dement. (2015) 11:1015–22. 10.1016/j.jalz.2015.04.011 26086182 PMC4581900

[166] Clare Morris M Tangney C Wang Y Sacks F Bennet D Aggarwal N MIND diet associated with reduced incidence of Alzheimer's disease. Alzheimer's Dement. (2015) 11:1007–14. 10.1016/j.jalz.2014.11.009 25681666 PMC4532650

[167] Männikkö R Komulainen P Schwab U Heikkilä HM Savonen K Hassinen M. The Nordic diet and cognition - The DR's EXTRA Study. Br J Nutr. (2015) 114:231–9. 10.1017/S0007114515001890 26104270

[168] Ngandu T Lehtisalo J Solomon A Levälahti E Ahtiluoto S Antikainen R. A 2 year multidomain intervention of diet, exercise, cognitive training, and vascular risk monitoring versus control to prevent cognitive decline in at-risk elderly people (FINGER): a randomised controlled trial. Lancet. (2015) 385:2255–63. 10.1016/S0140-6736(15)60461-5 25771249

[169] van Charante EPM Richard E Eurelings LS van Dalen JW Ligthart SA van Bussel EF Effectiveness of a 6-year multidomain vascular care intervention to prevent dementia (preDIVA): a cluster-randomised controlled trial. Lancet. (2016) 388:797–805. 10.1016/S0140-6736(16)30950-3 27474376

[170] Andrieu S Guyonnet S Coley N Cantet C Bonnefoy M Bordes S. Effect of long-term omega 3 polyunsaturated fatty acid supplementation with or without multidomain intervention on cognitive function in elderly adults with memory complaints (MAPT): a randomised, placebo-controlled trial. Lancet Neurol. (2017) 16:377–89. 10.1016/S1474-4422(17)30040-6 28359749

[171] Lam LC Chan WC Leung T Fung AW Leung EM. Would older adults with mild cognitive impairment adhere to and benefit from a structured lifestyle activity intervention to enhance cognition?: a cluster randomized controlled trial. PLoS ONE. (2015) 10:e0118173. 10.1371/journal.pone.0118173 25826620 PMC4380493

[172] Richard E Jongstra S Soininen H Brayne C Moll Van Charante EP Meiller Y. Healthy ageing through internet counselling in the elderly: the HATICE randomised controlled trial for the prevention of cardiovascular disease and cognitive impairment. BMJ Open. (2016) 6:1–10. 10.1136/bmjopen-2015-010806 27288376 PMC4908903

[173] Montero-Odasso M Almeida QJ Burhan AM Camicioli R Doyon J Fraser S. SYNERGIC TRIAL (SYNchronizing Exercises, Remedies in Gait and Cognition) a multi- Centre randomized controlled double blind trial to improve gait and cognition in mild cognitive impairment BMC Geriatr. (2018) 18:93. 10.1186/s12877-018-0782-7 29661156 PMC5902955

[174] Hardman RJ Kennedy G MacPherson H Scholey AB Pipingas A A randomised controlled trial investigating the effects of Mediterranean diet and aerobic exercise on cognition in cognitively healthy older people living independently within aged care facilities: the Lifestyle Intervention in Independent Living Aged Car. Nutr J. (2015) 14:1–10. 10.1186/s12937-015-0042-z 26003546 PMC4449609

[175] Daly RM Gianoudis J Prosser M Kidgell D Ellis KA O'Connell S. The effects of a protein enriched diet with lean red meat combined with a multi-modal exercise program on muscle and cognitive health and function in older adults: study protocol for a randomised controlled trial. Trials. (2015) 16:1–16. 10.1186/s13063-015-0884-x 26253520 PMC4529719

[176] Rovner B Casten R Hegel M Leiby B. Preventing cognitive decline in older african americans with mild cognitive impairment: design and methods of a randomized clinical tria. Contemp Clin Trials. (2012) 33:712–20. 10.1016/j.cct.2012.02.016 22406101 PMC3361551

[177] Kane RL Butler M Fink HA Interventions to Prevent Age-Related Cognitive Decline, Mild Cognitive Impairment, and Clinical Alzheimer's-Type Dementia Comparative Effectiveness Reviews. Rockville, MD: Agency for Healthcare Research and Quality (US). Report No.: 17-EHC008-EF (2017). 28759193

[178] Stern Y. Cognitive reserve in ageing and Alzheimer's disease. Lancet Neurol. (2012) 11:1006–12. 10.1016/S1474-4422(12)70191-6 23079557 PMC3507991

[179] Rakesh G Szabo S Alezopoulos G Zannas A. Strategies for dementia prevention: latest evidence and implications. Ther Adv Chronic Dis. (2017) 8:121–36. 10.1177/2040622317712442 28815009 PMC5546647

[180] Ramanathan A Nelson AR Sagare AP Zlokovic B V. Impaired vascular-mediated clearance of brain amyloid beta in Alzheimer's disease: the role, regulation and restoration of LRP1. Front Aging Neurosci. (2015) 7:1–12. 10.3389/fnagi.2015.00136 26236233 PMC4502358

[181] Vauzour D Camprubi-Robles M Miquel-Kergoat S Andres-Lacueva C Bánáti D Barberger-Gateau P. Nutrition for the ageing brain: towards evidence for an optimal diet. Ageing Res Rev. (2017) 35:222–40. 10.1016/j.arr.2016.09.010 27713095

[182] Hogervorst E Clifford A Stock J Xin X Bandelow S Exercise to prevent cognitive decline and alzheimer's disease: for whom, when, what, and (most importantly) how much? J Alzheimers Dis Park. (2012) 2:3 10.4172/2161-0460.1000e117

[183] Kivipelto M Solomon A Ahtiluoto S Ngandu T Lehtisalo J Antikainen R. The Finnish Geriatric Intervention Study to Prevent Cognitive Impairment and Disability (FINGER): study design and progress. Alzheimer's Dement. (2013) 9:657–65. 10.1016/j.jalz.2012.09.012 23332672

[184] Crous-Bou M Minguillón C Gramunt N Molinuevo JL. Alzheimer's disease prevention: from risk factors to early intervention. Alzheimers Res Ther. (2017) 9:71. 10.1186/s13195-017-0297-z 28899416 PMC5596480

[185] Rosenberg A Ngandu T Rusanen M Antikainen R Bäckman L Havulinna S. Multidomain lifestyle intervention benefits a large elderly population at risk for cognitive decline and dementia regardless of baseline characteristics: the FINGER trial. Alzheimer's Dement. (2018) 14:263–70. 10.1016/j.jalz.2017.09.006 29055814

[186] Zeevi D Korem T Zmora N Israeli D Rothschild D Weinberger A. Personalized nutrition by prediction of glycemic responses. Cell. (2015) 163:1079–95. 10.1016/j.cell.2015.11.001 26590418

[187] Celis-Morales C Livingstone KM Marsaux CFM Macready AL Fallaize R O'Donovan CB. Effect of personalized nutrition on health-related behaviour change: evidence from the Food4me European randomized controlled trial. Int J Epidemiol. (2016) 46:578–88. 10.1093/ije/dyw186 27524815

[188] Fallaize R Celis-Morales C MacReady AL Marsaux CF Forster H O'Donovan C. The effect of the apolipoprotein E genotype on response to personalized dietary advice intervention: findings from the Food4Me randomized controlled trial. Am J Clin Nutr. (2016) 104:827–36. 10.3945/ajcn.116.135012 27510539

[189] Celis-Morales C Marsaux CFM Livingstone KM Navas-Carretero S San-Cristobal R Fallaize R. Can genetic-based advice help you lose weight? Findings from the Food4Me European randomized controlled trial. Am J Clin Nutr. (2017) 105:1204–13. 10.3945/ajcn.116.145680 28381478

[190] Grimaldi KA van Ommen B Ordovas JM Parnell LD Mathers JC Bendik I. Proposed guidelines to evaluate scientific validity and evidence for genotype-based dietary advice. Genes Nutr. (2017) 12:35. 10.1186/s12263-017-0584-0 29270237 PMC5732517

[191] Pavlidis C Nebel J-C Katsila T Patrinos GP. Nutrigenomics 2.0: the need for ongoing and independent evaluation and synthesis of commercial nutrigenomics tests' scientific knowledge base for responsible innovation. Omi A J Integr Biol. (2016) 20:65–8. 10.1089/omi.2015.0170 26689492 PMC4770911

[192] Richard E Andrieu S Solomon A Mangialasche F Ahtiluoto S Van Charante EPM. Methodological challenges in designing dementia prevention trials - The European Dementia Prevention Initiative (EDPI). J Neurol Sci. (2012) 322:64–70. 10.1016/j.jns.2012.06.012 22818266

[193] Canevelli M Lucchini F Quarata F Bruno G Cesari M. Nutrition and dementia: evidence for preventive approaches? Nutrients. (2016) 8:1–10. 10.3390/nu8030144 26959055 PMC4808873

[194] Pan A Lin X Hemler E Hu FB. Diet and Cardiovascular disease: advances and challenges in population-based studies. Cell Metab. (2018) 27:489–96. 10.1016/j.cmet.2018.02.017 29514062 PMC5844273

[195] Hébert JR Frongillo EA Adams SA Turner-McGrievy GM Hurley TG Miller DR Perspective: randomized controlled trials are not a panacea for diet-related research. Adv Nutr. (2016) 7:423–32. 10.3945/an.115.011023 27184269 PMC4863268

[196] Satija A Yu E Willett WC Hu FB. Understanding nutritional epidemiology and its role in policy. Adv Nutr An Int Rev J. (2015) 6:5–18. 10.3945/an.114.007492 25593140 PMC4288279

[197] Laville M Segrestin B Alligier M Ruano-Rodríguez C Serra-Majem L Hiesmayr M. Evidence-based practice within nutrition: what are the barriers for improving the evidence and how can they be dealt with? Trials. (2017) 18:425. 10.1186/s13063-017-2160-8 28893297 PMC5594518

[198] Rollo ME Williams RL Burrows T Kirkpatrick SI Bucher T Collins CE What are they really eating? A review on new approaches to dietary intake assessment and validation. Curr Nutr Rep. (2016) 5:307–14. 10.1007/s13668-016-0182-6

[199] de Vries JHM de Groot LCPGM van Staveren WA. Dietary assessment in elderly people: experiences gained from studies in the Netherlands. Eur J Clin Nutr. (2009) 63:S69–74. 10.1038/ejcn.2008.68 19190649

[200] Day N McKeown N Wong M Welch A Bingham S. Epidemiological assessment of diet: a comparison of a 7-day diary with a food frequency questionnaire using urinary markers of nitrogen, potassium and sodium. Int J Epidemiol. (2001) 30:309–17. 10.1093/ije/30.2.309 11369735

[201] Carlsen MH Lillegaard IT Karlsen A Blomhoff R Drevon CA Andersen LF. Evaluation of energy and dietary intake estimates from a food frequency questionnaire using independent energy expenditure measurement and weighed food records. Nutr J. (2010) 9:1–9. 10.1186/1475-2891-9-37 20843361 PMC2949781

[202] Park Y Dodd KW Kipnis V Thompson FE Potischman N Schoeller DA. Comparison of self-reported dietary intakes from the Automated Self-Administered 24-h recall, 4-d food records, and food-frequency questionnaires against recovery biomarkers. Am J Clin Nutr. (2018) 107:80–93. 10.1093/ajcn/nqx002 29381789 PMC5972568

[203] Tooze JA Vitolins MZ Smith SL Arcury TA Davis CC Bell RA. High levels of low energy reporting on 24-hour recalls and three questionnaires in an elderly low-socioeconomic status population. J Nutr. (2007) 137:1286–93. 10.1093/jn/137.5.1286 17449594

[204] Andrieu S Coley N Lovestone S Aisen PS Vellas B. Prevention of sporadic Alzheimer's disease: lessons learned from clinical trials and future directions. Lancet Neurol. (2015) 14:926–44. 10.1016/S1474-4422(15)00153-2 26213339

[205] Johnson KA. Preclinical Alzheimer disease — the challenges ahead. Nat Rev Neurol. (2013) 9:54–8. 10.1038/nrneurol.2012.241 23183885 PMC3643203

[206] Schork NJ Goetz LH. Single-subject studies in translational nutrition research. Annu Rev Nutr. (2017) 37:395-422. 10.1146/annurev-nutr-071816-064717 28715990 PMC6383767

[207] Lillie EO Patay B Diamant J Issell B Topol EJ Schork NJ. The n-of-1 clinical trial: the ultimate strategy for individualizing medicine? Futur Med. (2012) 8:161–73. 10.2217/pme.11.7 21695041 PMC3118090

[208] Ferretti MT Iulita MF Cavedo E Chiesa PA Schumacher Dimech A Santuccione Chadha A. Sex differences in Alzheimer disease — the gateway to precision medicine. Nat Rev Neurol. (2018) 14:457–69. 10.1038/s41582-018-0032-9 29985474

[209] Adams PM Albert MS Albin RL Apostolova LG Arnold SE Asthana S Assessment of the genetic variance of late-onset Alzheimer's disease. Neurobiol Aging. (2016) 41:200.e13–20. 10.1016/j.neurobiolaging.2016.02.024 PMC4948179 27036079

[210] Gabler NB Duan N Liao D Elmore JG Ganiats TG Kravitz RL. Dealing with heterogeneity of treatment effects: is the literature up to the challenge? Trials. (2009) 10:43. 10.1186/1745-6215-10-43 19545379 PMC2706823

[211] Varadhan R Segal J Boyd C Wu A Weiss C. A framework for the analysis of heterogeneity of treatment effect in patient-centered outcomes research. J Clin Epidemiol. (2013) 66:818–25. 10.1016/j.jclinepi.2013.02.009 23651763 PMC4450361

[212] Whitlock EP Eder M Thompson JH Jonas DE Evans C V Guirguis-Blake JM. An approach to addressing subpopulation considerations in systematic reviews: the experience of reviewers supporting the U.S. Preventive Services Task Force. Syst Rev. (2017) 6:1–25. 10.1186/s13643-017-0437-3 28253915 PMC5335853

[213] Kent DM Hayward RA. Limitations of applying summary results of clinical trials to individual patients. The need for risk stratification. JAMA. (2007) 298:1209–12. 10.1001/jama.298.10.1209 17848656

[214] Kivipelto M Ngandu T Laatikainen T Winblad B Soininen H Tuomilehto J. Risk score for the prediction of dementia risk in 20 years among middle aged people: a longitudinal, population-based study. Lancet Neurol. (2006) 5:735–41. 10.1016/S1474-4422(06)70537-3 16914401

[215] Barnes DE Covinsky KE Whitmer RA Kuller LH Lopez OL Yaffe K. Predicting risk of dementia in older adults: the late-life dementia risk index. Neurology. (2009) 73:173–9. 10.1212/WNL.0b013e3181a81636 19439724 PMC2715571

[216] Mitchell S Ridley SH Sancho RM Norton M. The future of dementia risk reduction research: barriers and solutions. J Public Heal. (2017) 39:e275–81. 10.1093/pubmed/fdw103 27698267 PMC5896599

[217] Davidson KW Peacock J Kronish IM Edmondson D. Personalizing behavioral interventions through single-patient (N-of-1) trials. Soc Pers Psychol Compass. (2014) 8:408–21. 10.1111/spc3.12121 25267928 PMC4175746

[218] Kaput J Morine M. Discovery-based nutritional systems biology: developing N-of-1 nutrigenomic research. Int J Vitam Nutr Res. (2012) 82:333–41. 10.1024/0300-9831/a000128 23798052

[219] Isaacson RS. Is Alzheimer's prevention possible today? JAGS. (2017) 65:2153–4. 10.1111/jgs.15060 28846133 PMC5710839

[220] Ferron JM Moeyaert M Van den Noortgate W Natasha Beretvas S. Estimating causal effects from multiple-baseline studies: implications for design and analysis. Psychol Methods. (2014) 19:493–510. 10.1037/a0037038 24933294

[221] Levin JR Ferron JM Gafurov BS. Additional comparisons of randomization-test procedures for single-case multiple-baseline designs: alternative effect types. J Sch Psychol. (2017) 63:13–34. 10.1016/j.jsp.2017.02.003 28633936

[222] Lobo MA Moeyaert M Cunha AB Babik I. Single-case design, analysis, and quality assessment for intervention research. J Neurol Phys Ther. (2017) 41:187–97. 10.1097/NPT.0000000000000187 28628553 PMC5492992

[223] Tincani M Travers J Publishing single-case research design studies that do not demonstrate experimental control. Remedial Spec Educ. (2018) 39:118–28. 10.1177/0741932517697447

[224] McDonald S Quinn F Vieira R O'Brien N White M Johnston DW. The state of the art and future opportunities for using longitudinal n-of-1 methods in health behaviour research: a systematic literature overview. Health Psychol Rev. (2017) 11:307–23. 10.1080/17437199.2017.1316672 28406349

[225] Sharp DB Allman-Farinelli M. Feasibility and validity of mobile phones to assess dietary intake. Nutrition. (2014) 30:1257–66. 10.1016/j.nut.2014.02.020 24976425

[226] Forster H Walsh MC Gibney MJ Brennan L Gibney ER. Personalised nutrition: the role of new dietary assessment methods. Proc Nutr Soc. (2016) 75:96–105. 10.1017/S0029665115002086 26032731

[227] Gemming L Utter J Ni Mhurchu C. Image-assisted dietary assessment: a systematic review of the evidence. J Acad Nutr Diet. (2015) 115:64–77. 10.1016/j.jand.2014.09.015 25441955

[228] Guasch-Ferré M Bhupathiraju SN Hu FB. Use of metabolomics in improving assessment of dietary intake. Clin Chem. (2018) 64:82–98. 10.1373/clinchem.2017.272344 29038146 PMC5975233

[229] Wang Y Gapstur SM Carter BD Hartman TJ Stevens VL Gaudet MM. Untargeted metabolomics identifies novel potential biomarkers of habitual food intake in a cross-sectional study of postmenopausal women. J Nutr. (2018) 148:932–43. 10.1093/jn/nxy027 29767735

[230] Playdon MC Moore SC Derkach A Reedy J Subar AF Sampson JN. Identifying biomarkers of dietary patterns by using metabolomics. Am J Clin Nutr. (2017) 105:450–65. 10.3945/ajcn.116.144501 28031192 PMC5267308

[231] Garcia-Perez I Posma JM Gibson R Chambers ES Hansen TH Vestergaard H. Objective assessment of dietary patterns by use of metabolic phenotyping: a randomised, controlled, crossover trial. Lancet Diabetes Endocrinol. (2017) 5:184–95. 10.1016/S2213-8587(16)30419-3 28089709 PMC5357736

[232] Georgousopoulou EN Mellor DD Naumovski N Polychronopoulos E Tyrovolas S Piscopo S Mediterranean lifestyle and cardiovascular disease prevention. Cardiovasc Diagn Ther. (2017) 67:S39–47. 10.21037/cdt.2017.03.11 PMC5418209 28529921

[233] Ruano L Sousa A Severo M Alves I Colunas M Barreto R. Development of a self-administered web-based test for longitudinal cognitive assessment. Sci Rep. (2016) 6:1–10. 10.1038/srep19114 26743329 PMC4705487

[234] Duan N Kravitz RL Schmid CH. Single-patient (n-of-1) trials: a pragmatic clinical decision methodology for patient-centered comparative effectiveness research. J Clin Epidemiol. (2013) 66:S21–8. 10.1016/j.jclinepi.2013.04.006 23849149 PMC3972259

[235] Satija A Stampfer MJ Rimm EB Willett W Hu FB. Perspective: are large, simple trials the solution for nutrition research? Adv Nutr. (2018) 9:378–87. 10.1093/advances/nmy030 30032229 PMC6054238

[236] Wood WA Bennett A V Basch E. Emerging uses of patient generated health data in clinical research. Mol Oncol. (2014) 9:4–10. 10.1016/j.molonc.2014.08.006 25248998 PMC5528746

